# Performance evaluation of machine learning techniques in surface morphology and corrosion prediction for A286 3D printed micro-lattice structures

**DOI:** 10.1371/journal.pone.0320565

**Published:** 2025-05-07

**Authors:** Ameer Malik Shaik, Veera Siva Reddy B., Durga Prabhas S., Chandrasekhara Sastry C, J. Krishnaiah

**Affiliations:** 1 Combat Vehicles Research and Development Establishment (CVRDE), DRDO, Chennai, India; 2 Department of Mechanical Engineering (MED), Indian Institute of Information Technology Design and Manufacturing (IIITDM) Kurnoolhra Pradesh, India; Gazi Üniversitesi: Gazi Universitesi, TÜRKIYE

## Abstract

The development of lightweight, corrosion-resistant metallic lattice structures has gained significant attention in aerospace, defense, and structural applications, where material durability and weight optimization are critical. This study investigates the corrosion behavior of Laser Powder Bed Fusion (LPBF)-fabricated A286 steel honeycomb, Body-Centered Cubic (BCC), and gyroid lattices, comparing their performance against conventional materials such as Rolled Homogeneous Armor (RHA), Maraging High Strength Steel (MHA), and High-Nitrogen Steel (HNS). Corrosion testing was conducted using accelerated salt spray exposure, and the results were validated through computed tomography (CT)-based structural integrity analysis and machine learning-based predictive modeling. The experimental findings revealed that lattice structures exhibited significantly lower corrosion rates than conventional bulk materials, with the honeycomb lattice demonstrating the highest corrosion resistance (1.218 mm/year), followed by BCC (1.311 mm/year) and gyroid (1.671 mm/year). Compared to RHA, the honeycomb lattice exhibited a 57.23% reduction in corrosion rate, confirming its superior electrochemical stability. CT scan evaluations further highlighted differences in density distribution and geometric fidelity, with honeycomb lattices showing the most uniform porosity, while BCC structures displayed localized density variations at nodal intersections. To enhance predictive capabilities, various machine learning (ML) algorithms were employed to model corrosion behavior based on weight-loss measurements and lattice topology. Bayesian Ridge regression outperformed other models, achieving an R² of 0.99849 and RMSE of 0.00049, confirming its robustness in capturing corrosion trends. Linear Regression also performed well, while ensemble models such as Random Forest and XGBoost exhibited higher error margins due to dataset linearity constraints. Residual analysis and graphical interpretations further validated the stability and predictive reliability of ML-based corrosion assessments, demonstrating their feasibility as an alternative to traditional experimental methods. This study presents a comprehensive framework for integrating experimental corrosion testing, computational modeling, and CT-based defect analysis, offering a scalable approach to optimizing micro-lattice designs for corrosion-sensitive applications. The findings highlight the potential of LPBF-fabricated metallic lattices for aerospace, defense, and marine structures, where enhanced corrosion resistance, reduced material degradation, and predictive maintenance strategies are essential for long-term operational performance.

## Introduction

The increasing demand for high-performance materials in aerospace, defense, and advanced engineering applications has led to a paradigm shift toward lightweight, architected metallic structures [1]. Traditional metallic materials, such as rolled homogeneous armor (RHA), maraging high strength steel (MHA), and high-nitrogen steel (HNS), have been extensively utilized for their superior mechanical strength, wear resistance, and impact durability [2]. However, these materials often suffer from limitations in weight efficiency and corrosion resistance, particularly in extreme operational environments where exposure to chloride-rich atmospheres, high humidity, and fluctuating thermal conditions accelerates material degradation [3].

The advent of additive manufacturing (AM), particularly laser powder bed fusion (LPBF), has enabled the fabrication of intricate lattice structures that combine low density with high mechanical efficiency. Among these architectures, honeycomb, body-centered cubic (BCC), and gyroid lattices have gained prominence due to their unique load-bearing capacities, energy absorption characteristics, and potential corrosion resistance [4]. Despite these advantages, the corrosion susceptibility of such micro-lattice structures remains a critical concern, requiring a fundamental understanding of how lattice topology, surface exposure, and electrochemical interactions govern material degradation under corrosive conditions [5].

A key challenge in the study of LPBF-fabricated materials is their inherent microstructural heterogeneity, surface roughness, and porosity variations, all of which can influence corrosion behavior. Unlike bulk materials, lattice structures exhibit increased surface-area-to-volume ratios, which can either enhance corrosion resistance by promoting uniform passivation or exacerbate electrochemical degradation due to localized corrosion effects. While previous studies have explored the corrosion mechanisms of bulk AM-fabricated alloys, limited research has been conducted on the comparative corrosion resistance of different lattice topologies under identical environmental conditions. This study aims to address this gap by systematically evaluating honeycomb, BCC, and gyroid lattices, analyzing their corrosion performance relative to conventional materials, and integrating machine learning (ML) models to predict corrosion trends.

### Corrosion challenges in additively manufactured lattices

The corrosion behavior of metallic lattices is influenced by multiple interdependent factors, including surface topology, exposure to aggressive media, and electrochemical reaction kinetics [6]. In AM-fabricated structures, the rapid solidification process often leads to microstructural anisotropy, residual stresses, and oxide inclusions, which can significantly alter corrosion resistance [7]. Furthermore, the intricate nature of lattice architectures leads to non-uniform electrolyte penetration, making certain regions more vulnerable to localized pitting, crevice corrosion, and galvanic interactions [8].

Among the three studied lattice configurations, honeycomb lattices exhibit a more enclosed, columnar structure, which is expected to reduce electrolyte penetration and enhance corrosion resistance. BCC lattices, characterized by their nodal connectivity and open framework, may present increased surface exposure, leading to localized electrochemical activity. Gyroid structures, with their fully interconnected and curved surfaces, offer improved mechanical stability but may also provide higher active surface areas for electrochemical interactions, potentially increasing susceptibility to material loss. Understanding these relationships is crucial in determining the suitability of lattice structures for corrosion-sensitive applications, such as marine environments and aerospace components exposed to fluctuating humidity and saline conditions.

### Machine learning for corrosion rate prediction

Corrosion testing is conventionally performed using techniques such as weight-loss analysis, and accelerated salt spray exposure tests. While these methods provide valuable intuitions, they remain time-consuming, labor-intensive, and dependent on environmental fluctuations [9]. Machine learning-based predictive models have emerged as a powerful alternative to these traditional methodologies, enabling the development of real-time corrosion rate forecasting systems [10].

In this study, ML models were developed to predict corrosion rates based on experimental data, incorporating features such as initial and final weight measurements, total material loss, and lattice topology classifications. A range of ML models was explored, including Bayesian ridge regression, linear regression (LR), XGBoost, random forest (RF), and support vector regression (SVR). Each model was assessed based on its mean absolute error (MAE), root mean squared error (RMSE), and R² accuracy, ensuring a robust evaluation of predictive capabilities.

Bayesian Ridge and LR models were expected to perform well due to the strong linear correlation between total weight loss and corrosion rate. In contrast, tree-based models such as XGBoost and Random Forest, which rely on hierarchical feature splits, were hypothesized to show higher prediction variance due to the inherent linearity of the dataset. The ML-based predictive framework presented in this study provides a scalable approach for corrosion monitoring and optimization, reducing the need for prolonged experimental procedures.

### CT-based structural integrity analysis

Ensuring the dimensional accuracy and defect-free fabrication of metallic lattices is crucial for reliable long-term performance. Traditional methods of quality control, such as optical microscopy and SEM-based imaging, often provide only surface-level assessments, making it challenging to identify internal defects, porosity, or density variations in AM-fabricated components [11]. To overcome these limitations, Computed Tomography (CT) scanning was utilized in this study to non-destructively evaluate the internal structure of the fabricated lattices.

CT imaging enabled a quantitative assessment of density variations and geometric fidelity across honeycomb, BCC, and gyroid structures. The analysis confirmed that honeycomb lattices exhibited the most uniform porosity distribution, while BCC structures displayed localized density variations at nodal intersections, potentially affecting mechanical integrity and corrosion resistance. The gyroid lattice demonstrated a continuous surface network but exhibited minor fluctuations in grayscale intensity, suggesting slight density inconsistencies that could influence electrochemical performance. These findings provide a critical foundation for optimizing process parameters in LPBF fabrication and enhancing the structural consistency of corrosion-resistant lattice architectures.

This research advances the scientific understanding of corrosion-resistant lattice structures, demonstrating the synergistic potential of experimental, computational, and imaging techniques in material performance evaluation. By integrating salt spray testing, ML-based corrosion rate prediction, and CT-based defect analysis, this study provides a holistic approach to optimizing micro-lattice materials for real-world applications. The findings have significant implications for aerospace, defense, and marine industries, where corrosion-resistant, lightweight materials are paramount for operational efficiency and longevity.

## Materials and methods

### Material exploration

In modern engineering, the selection of materials for high-performance applications demands a balance between mechanical properties, weight, and cost-effectiveness. Materials such as composites, ceramics, and advanced alloys have gained prominence due to their unique characteristics [12]. Composite materials leverage the strengths of individual constituents to create a material with enhanced properties, but achieving uniformity in strength and properties remains a challenge. Additionally, their application often leads to an increase in overall weight, which may limit their efficiency in lightweight designs.

Ceramics like boron carbide and alumina are well-known for their exceptional toughness and hardness, making them suitable for applications requiring high wear resistance [13]. However, their inherent brittleness results in early failure under extensive loading, limiting their performance in dynamic conditions. On the other hand, advanced alloys such as titanium and aluminium alloys provide a favourable strength-to-weight ratio. Yet, they do not compare one-to-one with the performance of traditional steel in many contexts and often come with higher production costs, which can be a critical consideration in large-scale applications [14].

In the realm of steel, various grades are utilized for applications requiring durability and resilience. Examples include high-nitrogen steel (HNS), martensitic homogeneous steel (MHS), and rolled homogeneous steel (RHS). These materials exhibit distinct mechanical properties, with RHS being a high-strength low-alloy steel possessing tensile strength in the range of 910–1055 MPa and a hardness of 305 VHN. Martensitic homogeneous steel offers enhanced hardness (approximately 455 VHN) and higher tensile strength (1305–1455 MPa), making it suitable for demanding applications requiring impact resistance. HNS, with a tensile strength of 945–1080 MPa and a hardness of about 275 VHN, is particularly advantageous due to its cost-effectiveness and inherent non-magnetic properties [15].

Despite the favourable properties of these steels, several challenges remain in their use for advanced applications. A primary concern is the weight associated with high-thickness plates required for structural integrity and deformation resistance. This added weight can impact the efficiency and mobility of the final design and place additional stress on supporting components such as suspension systems. Another limitation lies in the stability and transportability of heavy structures, particularly in scenarios where space constraints or dynamic conditions come into play. Furthermore, the absence of powder forms of these materials limits their adaptability to modern manufacturing techniques such as laser powder bed fusion (LPBF), which restricts the ability to create advanced micro-lattice structures optimized for lightweight and high-strength applications.

To overcome these challenges, researchers are exploring alternative powder materials that can seamlessly integrate into the LPBF process, enabling the generation of modular solutions without compromising on hardness or structural integrity. The development of diverse micro-lattice structures, such as honeycomb, BCC and gyroid designs, provides a promising pathway for optimizing material performance. These lattice configurations are tailored to address issues related to weight and thickness while enhancing energy absorption, deformation resistance, and mechanical strength. The integration of advanced manufacturing processes with innovative material designs marks a significant step forward in creating solutions for high-performance engineering applications across various industries.

### Innovative micro lattice design framework

**Fig 1:**
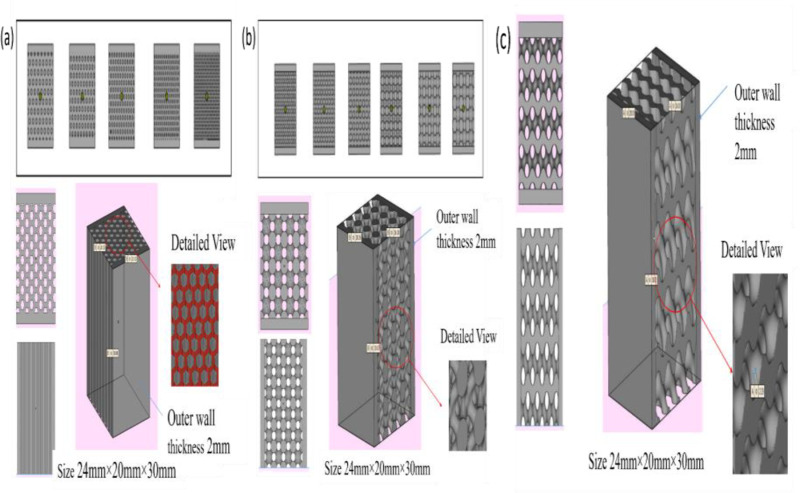
Micro lattice structure design variations and final test coupons (a) honeycomb (b) bcc, (c) gyroid.

The Incoloy alloy A286 steel exhibits properties comparable to high-performance structural materials, making it a strong candidate for applications requiring a combination of high strength and corrosion resistance [16]. In this study, honeycomb, BCC and gyroid lattice configurations were selected due to their superior mechanical performance and ability to reduce weight while maintaining structural integrity. The inherent properties of A286 steel, such as its excellent strength-to-weight ratio and resistance to corrosion, make it a versatile material for a wide range of advanced engineering applications. The final designs of the honeycomb and BCC test specimens were developed through a rigorous process of iterative optimization, aimed at achieving the best balance between weight reduction and mechanical performance. These designs, summarized in Table 1, were the result of systematic testing and refinement to ensure reliable material behaviour under diverse operational conditions. Fig 1 illustrates the honeycomb, BCC and gyroid lattice structures, which were precisely engineered to maximize energy absorption, deformation resistance, and structural stability.

**Table 1:** Micro lattice honeycomb and bcc structure design variations.

Gyroid	Design 1					
Dimension (L×W×H) (mm)	24×20×30					
X, Y, Z (mm) (Unit Cell)	5					
**Honeycomb**	**Design 1**	**Design 2**	**Design 3**	**Design 4**	**Design 5**	
Dimension (L×W×H) (mm)	24×20×30					
Hole Diameter and infill thickness (mm)	1.4	1.2	1	0.8	0.5	
**BCC Lattice**	**Design 1**	**Design 2**	**Design 3**	**Design 4**	**Design 5**	**Design 6**
Dimension (L×W×H) (mm)	24×20×30					
X, Y, Z (mm) (Unit Cell)	2	2.5	3	4	5	6
Ball Diameter (mm)	1.26	1.57	1.89	2.57	3.14	4.01

The iterative design process, similar to methodologies used for sandwich panels in aerospace and automotive industries, incorporated a 2mm outer thickness in the specimens. This feature was deliberately included to meet testing standards and provide enhanced support for applications involving high dynamic loads or impact conditions. The meticulous design and optimization of these lattice configurations highlight their potential for use in industries such as aerospace, automotive, and marine engineering, where lightweight and high-strength materials are critical for improved efficiency and performance. These findings emphasize the versatility of A286 steel and lattice structures in diverse applications, including lightweight structural components, energy absorption systems, and vibration damping solutions. By leveraging the inherent properties of A286 steel and the advanced capabilities of lattice configurations, this study contributes to the development of innovative material solutions for cutting-edge engineering challenges.

### Micro lattice fabrication through LPBF process

#### Characterizing raw materials.

The selection of Incoloy A286, a nickel and iron-based austenitic superalloy, is driven by its exceptional high-temperature strength and ability to withstand extreme stress conditions. These properties make A286 an ideal choice for advanced applications requiring superior mechanical performance under elevated temperatures, such as in aerospace, automotive, and industrial systems [17]. Its combination of strength, durability, and thermal stability offers significant advantages over conventional materials in challenging operating environments. A286 steel powders used for the fabrication of test coupons. The spherical shape of the powder is a critical attribute, ensuring smooth and uniform flow during the additive manufacturing process. It contributes to optimal packing density and precise layer formation, both of which are essential for achieving high-quality printed components. The chemical composition and physical properties of the material are detailed in Table 2.

**Table 2:** Properties of A286 steel.

Physical properties of A286 steel													
**Property**						**Value**							
Ultimate Tensile Strength						620 MPa							
Yield Strength						275 MPa							
Elongation at Break						40%							
Modulus of Elasticity						201 GPa							
Poisson’s Ratio						0.3							
Shear Modulus						77 GPa							
**Chemical composition (% weight factor) of A286 steel**													
**Element**	**C**	**Mn**	**Si**	**P**	**S**	**Ni**	**Cr**	**Mo**	**V**	**Al**	**Ti**	**Cu**	**Fe**
**Weight (%)**	0.034	0.126	0.236	0.012	0.006	25.62	15.40	1.331	0.196	0.209	2.054	0.014	Bal.

These characteristics of A286 steel make it particularly well-suited for applications where heat resistance, mechanical strength, and long-term reliability are paramount. Examples include high-performance turbine blades, exhaust manifolds, heat exchangers, and pressure vessels in industries such as aerospace, automotive, and power generation. The material’s excellent machinability and corrosion resistance further enhance its utility in environments involving high temperatures and corrosive elements, such as chemical processing plants and marine structures. By leveraging the advanced properties of A286 steel, this study contributes to a broader understanding of material performance in innovative lattice structures, highlighting its potential for use in diverse engineering applications that demand lightweight, high-strength, and thermally stable materials.

#### LPBF process.

**Fig 2:**
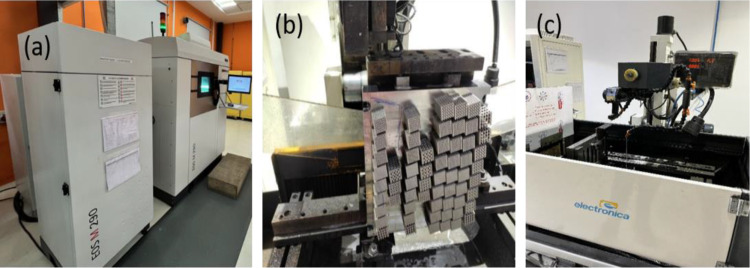
(a) LPBF process for printing of micro lattice structures: EOS M290 (b) Micro lattice structures (honeycomb & bcc) (c) w-EDM machine.

Fig 2a illustrates the EOS M290 laser powder bed fusion (LPBF) machine utilized for fabricating A286 steel micro-lattice specimens. These test specimens, incorporating integrated lattice structures, were manufactured using vacuum inert gas atomized A286 steel powders. The LPBF process employed a high-power incoherent Yb-fiber laser with a maximum output of 1000 W and a laser beam diameter of 75 µm. The machine features a build volume of 260 × 260 × 325 mm³, providing sufficient capacity to fabricate micro-lattice specimens with the desired dimensions for comparative analysis with conventional materials. During the manufacturing process, a uniform powder layer thickness of 20 µm was maintained to ensure precision and consistency.

Fig 2b displays the floor bed of the LPBF system along with the specimens in their as-printed condition. Following the printing process, wire electric discharge machining (W-EDM) was employed to carefully detach the specimens from the bottom support plate, as depicted in Fig 2c. This post-processing step ensured accurate removal of the test pieces without compromising their structural integrity.

#### Optimizing micro-lattice coupons: post-process treatment.

To optimize the mechanical properties of the micro-lattice coupons for applications requiring high strength and resilience, a rigorous post-process heat treatment protocol, based on the AMS 2773 standard, was employed. This multi-step treatment aimed to reduce residual stresses and refine the microstructure to enhance the material’s performance. The stress-relieved lattice structures were subjected to a solution treatment at temperatures ranging from 900°C to 1100°C for a minimum duration of 60 minutes. The specific temperature within this range was adjusted according to the exact chemical composition of the A286 steel powder to achieve the desired microstructural characteristics. This step facilitates the dissolution of alloying elements, homogenizing the material’s composition. Following the solution treatment, the specimens were rapidly quenched in oil to retain the high-temperature microstructure and prevent undesirable phase transformations. This was followed by a precipitation hardening process conducted at a temperature between 680°C and 750°C for 16 hours, with the final step being air cooling. This precipitation hardening phase promotes the formation of fine precipitates within the microstructure, which significantly enhance the material’s strength and hardness.

The comprehensive heat treatment regimen was meticulously analysed to evaluate its effects on tensile residual stress reduction, microstructural refinement, and mechanical property optimization. This process demonstrated a notable improvement in the strength and hardness of the micro-lattices, making them suitable for a wide range of high-performance applications [18]. These include aerospace components such as turbine blades and structural panels, automotive parts requiring lightweight yet robust materials, and industrial applications such as heat exchangers and high-pressure vessels. The insights gained from this study contribute to a deeper understanding of the interplay between heat treatment parameters and material performance, providing a pathway for the development of advanced lattice structures for engineering applications that demand superior strength, thermal stability, and reliability under dynamic and static loading conditions.

### Evaluating LPBF-manufactured micro-lattice structures

The evaluation of LPBF-manufactured micro-lattice structures is crucial for understanding their mechanical performance and suitability for structural applications. This study examines three distinct lattice architectures honeycomb, BCC, and gyroid each possessing unique geometric characteristics that influence mechanical properties such as strength, toughness, and failure mechanisms under different loading conditions.

The honeycomb lattice, with its interconnected hexagonal cells, exhibits high in-plane stiffness and energy absorption capabilities, making it well-suited for applications requiring impact resistance and crashworthiness. The BCC lattice, in contrast, consists of nodal intersections that distribute loads efficiently, but its open-cell structure increases susceptibility to localized shear failure under certain stress conditions.

The gyroid lattice, a triply periodic minimal surface (TPMS) structure, offers a continuous, smooth surface architecture that reduces stress concentrations while maintaining isotropic mechanical properties. Unlike the anisotropic stiffness observed in honeycomb and BCC lattices, the gyroid structure enables a more uniform stress distribution, making it beneficial for applications requiring balanced mechanical performance across multiple loading directions. Its fully interconnected network also enhances load transfer efficiency, reducing localized weak points that might contribute to early failure.

Due to their distinct architectural differences, the selection of a lattice structure depends on the specific mechanical requirements of the intended application. The honeycomb lattice is ideal for applications needing high strength and energy dissipation, the BCC lattice balances weight reduction with reasonable structural integrity, and the gyroid lattice provides an optimal combination of load distribution and isotropic strength, making it suitable for fluid flow applications, thermal management systems, and multifunctional structural components.

Surface roughness and micro-Vickers hardness are critical parameters in evaluating the mechanical properties of these lattice structures. Surface roughness, which defines the texture and quality of the material’s surface, plays a vital role in determining performance under dynamic loading conditions. A rough surface can create localized stress concentrations, potentially leading to premature failure under repetitive or high-intensity loads. Thus, minimizing surface roughness is essential to enhance the material’s durability and reliability. Surface roughness measurements were conducted using an MFT-5000 Tribometer (RTEC Instruments) with an R-20x lens and white light. The data acquired were post-processed using the Gwyddion software (64-bit) to obtain average surface roughness values at specified locations. These evaluations provided insights into the surface quality of the lattice structures, contributing to a better understanding of their manufacturing consistency and structural integrity.

Micro-Vickers hardness is another critical factor in assessing the material’s mechanical performance. It measures the resistance of the lattice structure to deformation and penetration under static or dynamic loads. By selecting appropriate micro-Vickers hardness values, the mechanical strength of the structures can be optimized to meet the demands of various applications. The micro-hardness of the LPBF-manufactured lattice specimens was evaluated following ISO 14577–1 standards using the Tukon/Wilson 1102 Vickers hardness tester (scale: microns), with load options ranging from 0.01 to 2 kg. To ensure statistical reliability, six measurements were taken per sample, with multiple indentations performed on the same surface due to the intricate geometries of the lattice structures.

The study analysed LPBF-manufactured coupons under three conditions: as-printed, stress-relieved, and heat-treated. These conditions were evaluated for both honeycomb, BCC, and gyroid lattice structures to understand how processing techniques influence surface roughness and hardness. Response variables related to each design criterion for honeycomb, bcc and gyroid lattices are presented in Table 3–5. Each set of final heat-treated coupons consisted of three replicates, coded uniquely for systematic analysis. This comprehensive evaluation highlights the potential of these lattice structures in applications such as aerospace components, automotive crash absorption systems, industrial vibration dampers, and energy-efficient structural elements.

**Table 3:** Response variables, surface roughness and micro-Vickers hardness for honeycomb micro-lattice structure.

As printed									
S. No.	Lattice	Design	Non-Stress Relieved	Surface roughness (Ra)			Micro Vickers Hardness (HV)		
				Average (Ra)	Max	Min	Average	Max	Min
1	Honeycomb	Design 1	2A	11.29	11.64	10.54	219 HV 1	225	213
2		Design 2	3A	9.94	11.84	9.36	220 HV 1	227	215
3		Design 3	4A	11.18	11.18	9.87	230 HV 1	242	218
4		Design 4	5A	8.91	12.6	8.54	213 HV 1	218	207
5		Design 5	6A	11.69	12.34	9.03	194 HV 1	201	190
**Stress relieved**									
**S. No.**	**Lattice**	**Design**	**Stress Relieved**	**Average (Ra)**	**Max**	**Min**	**Average**	**Max**	**Min**
1	Honeycomb	Design 1	2B	14.58	24.26	9.23	233 HV 1	239	228
2		Design 2	3B	14.79	17.12	10.48	214 HV 1	220	209
3		Design 3	4B	13.76	15.91	12.11	193 HV 1	197	190
4		Design 4	5B	12.26	13.66	11.43	222 HV 1	226	218
5		Design 5	6B	20.24	24.23	17.19	220 HV 1	226	216
**Heat Treated**									
**S. No.**	**Lattice**	**Design**	**Heat Treated**	**Average (Ra)**	**Max**	**Min**	**Average**	**Max**	**Min**
1	Honeycomb	Design 1	2C,2D,2E	11.59	15.2	9.12	460 HV 1	475	451
2		Design 2	3C,3D.3E	7.77	7.94	7.65	418 HV 1	425	412
3		Design 3	4C,4D,4E	7.67	8.46	6.63	423 HV 1	426	420
4		Design 4	5C,5D,5E	8.82	8.87	8.77	460 HV 1	470	451
5		Design 5	6C.6D,6E	11.92	12.81	10.41	455 HV 1	461	450

**Table 4:** Response variables, surface roughness and micro-Vickers hardness for bcc micro-lattice structure.

S. No.	Lattice	Design	Non-Stress Relieved	Surface roughness (Ra)			Micro Vickers Hardness (HV)		
				Average (Ra)	Max	Min	Average	Max	Min
**As Printed**									
1	BCC	Design 1	7A	14.7	14.7	11.56	194 HV 1	196	190
2		Design 2	8A	14.34	14.3	10.56	199 HV 1	204	196
3		Design 3	9A	12.31	12.97	10.21	215 HV 1	221	207
4		Design 4	10A	12.04	13.29	11.3	217 HV 1	222	212
5		Design 5	11A	10.54	12.52	10.03	229 HV 1	235	226
6		Design 6	12A	10.61	12.34	10	222	2	218
**Stress relieved**									
**S. No.**	**Lattice**	**Design**	**Heat Treated**	**Average (Ra)**	**Max**	**Min**	**Average**	**Max**	**Min**
1	BCC	Design 1	7B	15.7	16.92	14.9	214 HV 1	218	211
2		Design 2	8B	11.44	13.56	8.85	219 HV 1	224	214
3		Design 3	9B	10.65	12.41	8.95	232 HV 1	235	227
4		Design 4	10B	10.33	10.89	9.48	243 HV 1	245	240
5		Design 5	11B	7.84	8.82	6.4	255 HV 1	259	249
6		Design 6	12B	9.92	11.18	7.55	229 HV 1	235	221
**Heat Treated**									
1	BCC	Design 1	7C,7D,7E	7.31	7.82	6.79	389 HV 1	393	386
2		Design 2	8C,8D,8E	6.47	6.67	6.29	398 HV 1	402	395
3		Design 3	9C,9D,9E	7.91	8.6	7.36	398 HV 1	399	396
4		Design 4	10C,10D,10E	6.82	7.51	6.2	400 HV 1	405	394
5		Design 5	11C,11D,11E	4.45	5.46	2.87	411 HV 1	416	409
6		Design 6	12C,12D,12E	5.42	5.56	5.27	368 HV 1	377	361

**Table 5:** Response variables, surface roughness and micro-Vickers hardness for gyroid micro-lattice structure.

Non-stress relived (As printed)									
S. No.	Lattice	Design	Non-Stress Relieved	Surface roughness (Ra)			Micro Vickers Hardness (HV)		
				Average (Ra)	Max	Min	Average	Max	Min
1	Gyroid	Design 1	1A	10.5	12.86	8.11	215 HV 1	211	220
**Stress relieved**									
**S. No.**	**Lattice**	**Design**	**Stress Relieved**	**Average (Ra)**	**Max**	**Min**	**Average**	**Max**	**Min**
1	Gyroid	Design 1	1B	11.85	13.46	10.83	226 HV 1	222	229
**Heat Treated (Final specimens)**									
**S. No.**	**Lattice**	**Design**	**Heat Treated**	**Average (Ra)**	**Max**	**Min**	**Average**	**Max**	**Min**
1	Gyroid	Design 1	1C,1D,1E	3.56	3.74	3.37	418 HV 1	411	425

By integrating the insights from surface roughness and micro-hardness evaluations, this study contributes to the broader understanding of optimizing LPBF-manufactured lattice structures for applications requiring lightweight, durable, and high-strength materials. The findings underscore the versatility of these structures in addressing challenges in diverse engineering domains, paving the way for innovations in material design and additive manufacturing.

The lattice structures demonstrated substantial improvements in surface characteristics and mechanical properties following a multi-step heat treatment process. Compared to the as-printed condition, surface roughness was reduced significantly, ranging from 35.74% to 57.77% for BCC structures, 14.14% to 21.83% for honeycomb structures, and 20.32% to 29.48% for gyroid structures. The reduction in surface roughness is attributed to grain boundary migration and surface diffusion during heat treatment, which facilitates void filling and atomic rearrangement, leading to a smoother surface. The gyroid lattice exhibited a moderate reduction in roughness, benefiting from its continuous TPMS geometry, which enables more uniform diffusion compared to BCC and honeycomb structures.

Hardness improvements followed a similar trend, increasing by 39.67% to 50.12% for BCC structures, 45.62% to 57.36% for honeycomb structures, and 34.89% to 42.71% for gyroid structures. The enhancement in hardness is primarily due to the formation of a refined grain structure during heat treatment, which strengthens the lattice by reducing dislocation movement. The gyroid lattice exhibited a slightly lower increase in hardness, as its fully interconnected structure distributes stress more evenly, reducing localized strengthening effects observed in honeycomb and BCC lattices.

To determine the ideal dimensions and properties of the test coupons, a statistical approach was employed. This method identified the optimal vector plane satisfying the conditions for maximum hardness and minimal surface roughness. The analysis accounted for various factors and responses, ensuring that the chosen configurations are suitable for applications requiring lightweight, strong, and durable materials. The findings have significant implications for a wide range of applications, including aerospace components such as structural supports and turbine blades, automotive systems requiring crash-resistant lightweight materials, and industrial equipment subjected to high stress and wear. The integration of heat treatment with LPBF fabrication highlights the potential for advancing material performance, offering innovative solutions for engineering challenges in diverse industries. By achieving a balance between surface characteristics and mechanical properties, this study contributes to the broader understanding of optimizing lattice structures for high-performance applications.

#### The statistical evolution method for micro-lattice structures: MCDM TOPSIS.

Multi-criteria decision-making (MCDM) is a sophisticated discipline that empowers decision-makers to evaluate and prioritize alternatives based on multiple attributes simultaneously. It integrates various statistical methods to systematically analyse alternatives across diverse criteria, facilitating informed decision-making in complex scenarios. Among the prominent statistical techniques used in MCDM are the Analytic Hierarchy Process (AHP), the Technique for Order of Preference by Similarity to Ideal Solution (TOPSIS), and the Preference Ranking Organization Method for Enrichment Evaluations (PROMETHEE) [19]. Each method provides unique benefits, including the ability to rank alternatives, analyse trade-offs between competing criteria, and offer a structured framework for tackling multifaceted decision problems. These techniques have found widespread application in areas requiring material selection, design optimization, and other engineering domains where a comprehensive evaluation of alternatives is crucial.

Among these methods, TOPSIS stands out for its relative simplicity, computational efficiency, and versatility. It is particularly effective in handling both quantitative and qualitative criteria, offering clear and interpretable results. The core principle of TOPSIS lies in identifying an alternative that is closest to the ideal solution while being farthest from the negative ideal solution. This dual consideration provides a balanced perspective, making it a popular choice in material evaluation and design optimization. The methodology involves normalizing criteria values to eliminate dimensional bias, weighting them to reflect their relative importance, and calculating the separation distance of each alternative from the ideal and negative ideal solutions. This process culminates in a ranking that highlights the most suitable alternative based on the defined criteria.

TOPSIS has been extensively applied in material science and engineering for optimizing material properties, selecting lightweight and high-strength materials for structural components, and evaluating the suitability of coatings and composites for energy-efficient systems. Its adaptability makes it a valuable tool in advanced manufacturing techniques such as laser powder bed fusion (LPBF), where multiple performance parameters must be simultaneously optimized. By balancing conflicting criteria like strength, weight, and surface quality, TOPSIS enables researchers and engineers to make data-driven decisions that enhance the performance and reliability of engineered components.

This study leverages TOPSIS to evaluate and rank LPBF-fabricated lattice structures for their mechanical performance and surface characteristics. By employing a structured approach, this research offers critical insights into selecting optimal lattice configurations for a wide range of applications, including aerospace, automotive, and industrial systems. The outcomes demonstrate how MCDM techniques, particularly TOPSIS, can drive innovation in material design and foster advancements in engineering solutions tailored to specific performance requirements.

1 Normalization of performance

Normalized performance of alternative $a_{i}$ on criterion $b_{j}$ is determined by:


$$r_{ij}=\frac{a_{ij}}{\sqrt{\sum_{k=1}^{n}\;\;b_{kj}^{2}}}$$ (1)

2 Weight allocation:

Weight $c_{j}$ allocated to criterion ${\;d}_{j}$, where $c_{j}\geq 0$ and ${\;\sum }_{j=1}^{m}\;c_{j}=1$.

3 Decision matrix construction:

Construct a matrix$\;X=\left[r_{ij}\right]$, where rows represent alternatives and columns represent criteria.

4 Ideal and negative ideal solutions:

- Ideal solution$Y^{+}=\left(y_{1}^{+},y_{2}^{+},\ldots ,y_{m}^{+}\right)$, where$y_{j}^{+}=max\left(r_{ij}\cdot c_{j}\right)$., where n and m are the number of rows and columns, respectively.
- Negative ideal solution $Y^{-}=\left(y_{1}^{-},y_{2}^{-},\ldots ,y_{m}^{-}\right)$ is determined by $y_{\bar{j}}^{-}=min\left(r_{ij}\cdot c_{j}\right)$ for all alternatives  i=1,2,…,n.

5 Euclidean distance:

$$S_{i}^{+}=\sqrt{\sum_{j=1}^{m}\;\;c_{j}\cdot {\left(r_{ij}-y_{j}^{+}\right)}^{2}}$$ (2)

- Distance of $x_{i}$ from ideal solution:
- Distance of $e_{i}$ from negative ideal solution:

$$\begin{array}{c} S_{i}^{-}=\sqrt{\sum_{j=1}^{m}\;\;c_{j}\cdot {\left(r_{ij}-y_{j}^{-}\right)}^{2}} \end{array}$$ (3)

6 Relative proximity

$$Z_{i}=\frac{S_{i}^{-}}{S_{i}^{+}+S_{i}^{-}}$$ (4)

Relative closeness of $e_{i}$ to ideal solution:

7 The alternatives should be ranked according to their proximity to the ideal solution.

- The alternative that exhibits the greatest degree of relative closeness is deemed to be the optimal alternative.

The application of this analytical interpretive approach results in a TOPSIS analysis for honeycomb and bcc structures corresponding to as printed, stress relieved, and trailing by heat treatment condition as depicted in Table 6 and 7.

**Table 6:** TOPSIS analysis for honeycomb micro-lattice structure for LPBF-generated coupons.

As printed								
**Normalized Residual Matrix**			**Weighted Residual Matrix**		**Distance to Ideal**			
**Coupon no.**	**NRa**	**NVHN**	**WRa**	**WVHN**	**Si+**	**Si-**	**cci**	**Rank**
2A	0.369	0.419	0.185	0.209	0.039	0.007	0.154	4
3A	0.325	0.421	0.163	0.210	0.017	0.029	0.633	2
4A	0.365	0.440	0.183	0.220	0.037	0.010	0.204	3
5A	0.291	0.408	0.146	0.204	0.000	0.046	0.994	1
6A	0.382	0.371	0.191	0.186	0.047	0	0	5
**Stress relieved**								
**Normalized Residual Matrix**			**Weighted Residual Matrix**		**Distance to Ideal**			
**Coupon no.**	**NRa**	**NVHN**	**WRa**	**WVHN**	**Si+**	**Si-**	**cci**	**Rank**
2B	0.477	0.446	0.239	0.223	0.038	0.094	0.713	3
3B	0.484	0.409	0.242	0.205	0.042	0.090	0.682	4
4B	0.450	0.369	0.225	0.185	0.026	0.106	0.803	2
5B	0.401	0.425	0.201	0.212	0.000	0.131	0.999	1
6B	0.662	0.421	0.331	0.210	0.131	0.001	0.005	5
**Heat treated**								
**Normalized Residual Matrix**			**Weighted Residual Matrix**		**Distance to Ideal**			
**Coupon no.**	**NRa**	**NVHN**	**WRa**	**WVHN**	**Si+**	**Si-**	**cci**	**Rank**
2C,2D,2E	0.379	0.880	0.190	0.440	0.064	0.007	0.099	4
3C,3D.3E	0.254	0.800	0.127	0.400	0.003	0.068	0.954	2
4C,4D,4E	0.251	0.809	0.125	0.405	0.001	0.070	0.982	1
5C,5D,5E	0.289	0.880	0.144	0.440	0.019	0.052	0.736	3
6C.6D,6E	0.390	0.871	0.195	0.435	0.070	0.001	0.018	5

**Table 7:** TOPSIS analysis for bcc micro-lattice structure for LPBF-generated coupons.

As printed								
**Normalized Residual Matrix**			**Weighted Residual Matrix**		**Distance to Ideal**			
**Coupon no.**	**NRa**	**NVHN**	**WRa**	**WVHN**	**Si+**	**Si-**	**cci**	**Rank**
7A	0.481	0.371	0.241	0.186	0.069	0.000	0.000	6
8A	0.469	0.381	0.235	0.190	0.063	0.006	0.086	5
9A	0.403	0.411	0.201	0.206	0.029	0.040	0.576	4
10A	0.394	0.415	0.197	0.208	0.025	0.044	0.641	3
11A	0.345	0.438	0.172	0.219	0.000	0.069	1	1
12A	0.347	0.425	0.174	0.212	0.001	0.068	0.983	2
**Stress relieved**								
**Normalized Residual Matrix**			**Weighted Residual Matrix**		**Distance to Ideal**			
**Coupon no.**	**NRa**	**NVHN**	**WRa**	**WVHN**	**Si+**	**Si-**	**cci**	**Rank**
7A	0.514	0.409	0.257	0.205	0.130	0	0	6
8A	0.374	0.419	0.187	0.209	0.060	0.070	0.537	5
9A	0.348	0.444	0.174	0.222	0.046	0.083	0.641	4
10A	0.338	0.465	0.169	0.232	0.041	0.089	0.684	3
11A	0.257	0.488	0.128	0.244	0	0.130	1	1
12A	0.325	0.438	0.162	0.219	0.035	0.095	0.732	2
**Heat treated**								
**Normalized Residual Matrix**			**Weighted Residual Matrix**		**Distance to Ideal**			
**Coupon no.**	**NRa**	**NVHN**	**WRa**	**WVHN**	**Si+**	**Si-**	**cci**	**Rank**
7A	0.24	0.74	0.120	0.372	0.047	0.010	0.178	5
8A	0.21	0.76	0.106	0.381	0.033	0.024	0.423	3
9A	0.26	0.76	0.129	0.381	0.057	0.001	0.014	6
10A	0.22	0.77	0.112	0.383	0.039	0.019	0.326	4
11A	0.15	0.79	0.073	0.393	0	0.058	1	1
12A	0.18	0.70	0.089	0.352	0.018	0.041	0.699	2

Using the statistical method TOPSIS, the close-to-ideal solution for A286 micro-lattice structures was determined under various post-processing conditions. For honeycomb specimens in as-printed and stress-relieved states, the optimal dimensions were identified as 24 mm × 20 mm × 30 mm (L×W×H), with a wall thickness of 2 mm, a hole diameter of 0.8 mm, and an infill thickness of 0.8 mm. For heat-treated (final) specimens, the overall external dimensions remained unchanged, but internal geometric parameters showed refinement with a hole diameter and infill thickness of 1 mm. In comparison, the optimal configurations for BCC A286 micro-lattice structures under as-printed, stress-relieved, and heat-treated conditions were consistent, with external dimensions of 24 mm × 20 mm × 30 mm (L×W×H) and unit cell dimensions of 5 mm × 5 mm × 5 mm (X×Y×Z).

Since TOPSIS was not applied to the gyroid lattice, its evaluation was instead based on dimensional consistency and geometric integrity across different post-processing conditions. The gyroid lattice maintained its design fidelity with minimal geometric distortions, owing to its continuous surface topology, which promotes uniform shrinkage and stress redistribution during heat treatment. Unlike the discrete nodal intersections in BCC and honeycomb lattices, the gyroid’s self-supporting structure exhibited greater resistance to localized deformation, ensuring stable dimensional accuracy post-processing. This characteristic makes gyroid lattices particularly suitable for applications requiring structural uniformity and isotropic mechanical properties.

#### Corrosion analysis via salt spray testing.

**Fig 3:**
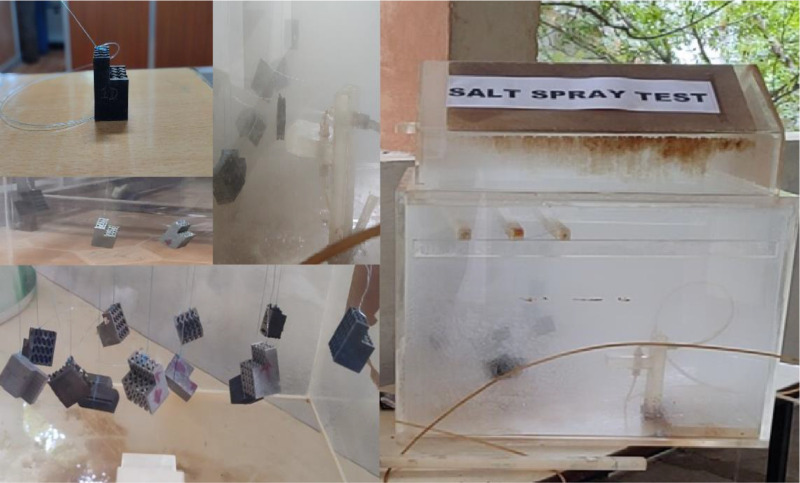
Salt spray test setup and sample exposure.

The corrosion resistance of the BCC, honeycomb, and gyroid lattice structures, along with conventional bulk materials, was evaluated using salt spray testing (ASTM B117 standard) to simulate accelerated corrosive environmental conditions. The test was conducted in a controlled salt spray chamber, as shown in Fig 3, where specimens were exposed to a fine mist of 5 wt.% NaCl solution at an operating temperature of 35 ± 2°C. This method provides a standardized assessment of material degradation, surface oxidation, and weight loss due to corrosion.

The experiment included BCC, honeycomb in both ideal and non-ideal configurations, and gyroid lattice along with conventional materials such as RHA, MHA, and HNS. Each type of structure was tested under different conditions, including as-printed, stress-relieved, and heat-treated states, ensuring a comprehensive evaluation of their corrosion performance.

A total of six specimens were tested for each category of the honeycomb lattice, covering its ideal and non-ideal designs, while the gyroid and BCC lattices were represented by three specimens each for the same processing conditions. For comparison, one specimen each of RHA, MHA, and HNS was also subjected to the same corrosion exposure conditions.

Each specimen was suspended in the chamber using non-corrosive polymeric wires to prevent any external contamination or electrochemical interaction with the chamber walls. The samples were exposed to the salt spray in two cycles, each lasting 24 hours, followed by a 24-hour atmospheric drying period before repeating the process. This resulted in a total exposure duration of 48 hours, allowing for the formation of corrosion products, surface pitting, and potential localized degradation.

After completing the exposure cycles, the specimens were carefully removed from the chamber and subjected to a standardized cleaning process. Initially, the samples were rinsed with deionized water to remove any loosely attached salt deposits. This was followed by a two-step chemical cleaning process using acetone and methanol/ethanol, ensuring that any residual contaminants or oxidation layers were effectively eliminated before final weight measurements.

The corrosion rate was determined by measuring the weight reduction of each specimen before and after exposure. The weight measurements were conducted using a high-precision analytical balance (± 0.1 mg accuracy), ensuring reliable data collection. The corrosion rate was then calculated using the corrosion rate equation


$$Corrosion\;Rate\left(\frac{mm}{year}\right)=\frac{K\times \Delta W}{A\times T\times D}$$

Where K is the 8.76×104 (constant for unit conversion), ∆W is weight loss in grams, A is the exposed surface area in cm², T is the exposure duration in hours, D is the material density in g/cm³.

This calculation provides a quantitative assessment of the material’s corrosion susceptibility, enabling a direct comparison between micro-lattice structures and conventional bulk materials [20]. The corrosion rates derived from these tests are crucial in understanding the degradation mechanisms associated with different lattice configurations, particularly in high-performance applications where material longevity is a key factor.

## Results and discussion

### CT scan analysis of lattice structures

Computed tomography (CT) imaging provides a non-destructive evaluation of the internal structure and integrity of the metallic micro-lattice designs. The gyroid, honeycomb, and BCC lattice structures were scanned to assess their internal morphology, density distribution, and potential manufacturing defects. The resulting CT images allow for a quantitative assessment of lattice uniformity, porosity variations, and defect formation, which are critical parameters influencing mechanical performance and corrosion resistance [21].

Each lattice was analyzed based on cross-sectional views, grayscale intensity variations, and dimensional accuracy extracted from CT scan data. The grayscale intensity represents material density distribution, where higher intensity values correspond to denser regions, and lower values indicate porous or defect-prone areas.

#### CT scan evaluation of gyroid lattice.

**Fig 4:**
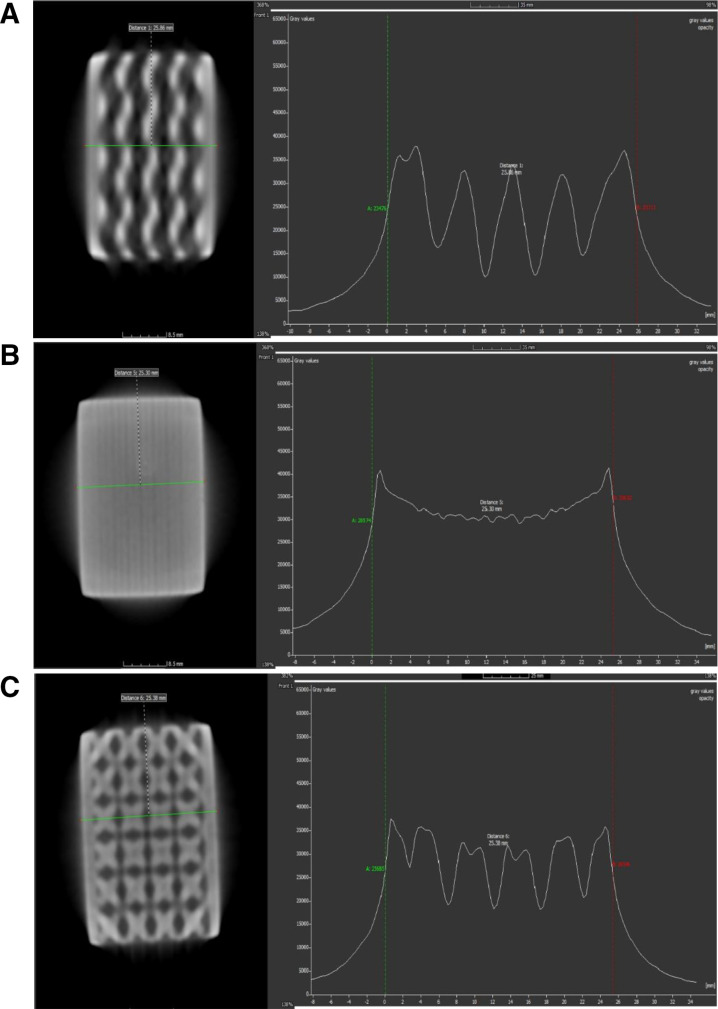
(a) CT scan analysis of gyroid lattice with nodal density distribution. (b) CT scan analysis of honeycomb lattice with nodal density distribution. (c) CT Scan analysis of BCC lattice with nodal density distribution.

The CT scan of the gyroid lattice structure is shown in Fig 4a. The top, front, and right sectional views reveal a highly periodic interconnected structure, characteristic of a triply periodic minimal surface (TPMS). The density variations observed in the grayscale intensity plot confirm the presence of well-distributed material with periodic density fluctuations.

The distance measurement along the vertical axis indicates a uniform cell spacing of approximately 25.38 mm, aligning well with the designed lattice parameters. The grayscale intensity profile in Fig 4a exhibits a periodic pattern, indicating a consistent material distribution. The fluctuations in intensity are attributed to the curved nature of the gyroid surfaces, which vary in thickness across different cross-sections.

These results confirm that the gyroid structure maintains high geometric fidelity, with minimal defects or irregularities detected in the CT scan. The well-distributed internal porosity suggests potential advantages in mechanical energy absorption and fluid flow characteristics, making it a promising candidate for lightweight structural and filtration applications.

#### CT scan evaluation of honeycomb lattice.

The honeycomb lattice structure, depicted in Fig 4b, exhibits a distinct layered architecture with anisotropic porosity distribution. Unlike the gyroid lattice, which features a continuous, interconnected framework, the honeycomb structure presents stacked, columnar cells.

The top-sectional view highlights a hexagonal arrangement, maintaining consistent cell spacing (25.30 mm) along the vertical axis, as confirmed by the CT scan measurements. The front and side projections illustrate a denser material distribution in the horizontal planes, suggesting anisotropic stiffness characteristics.

The grayscale intensity plot (Fig 4b) reveals a gradual increase in density across the structure, reflecting the thicker walls and reinforcement at specific cross-sections. This characteristic enhances load-bearing capacity along the primary axis, making honeycomb structures highly suitable for impact energy absorption and mechanical reinforcement applications.

Overall, the CT analysis confirms that the honeycomb lattice maintains dimensional stability with uniform cell spacing, but also exhibits directional variations in material density, which can influence its mechanical anisotropy and failure behavior under different loading conditions.

#### CT scan evaluation of BCC lattice.

The BCC lattice structure, shown in Fig 4c, presents a highly porous architecture with well-defined nodal intersections. The top view highlights a repeating cubic network, forming a framework with interconnected nodes that enhances its load-bearing efficiency and energy dissipation capabilities.

From the vertical sectional analysis, the measured cell spacing is approximately 25.36 mm, demonstrating good fabrication accuracy relative to the designed dimensions. The front and side views indicate a variation in material density at the nodal intersections, where stress concentrations are typically higher.

The grayscale intensity profile (Fig 4c) shows periodic peaks corresponding to the nodal intersections, confirming non-uniform density distribution, with higher material concentration at connection points. These characteristics make BCC lattices particularly efficient for weight reduction while maintaining structural stability.

Despite the well-formed nodal connectivity, minor variations in grayscale intensity suggest possible porosity inconsistencies at specific sections, which could affect its mechanical performance under dynamic loading conditions. This finding emphasizes the importance of process parameter optimization during fabrication, ensuring uniform density across the entire lattice structure.

#### Comparative analysis of CT scan results.

The CT analysis of the gyroid, honeycomb, and BCC lattices reveals distinct differences in internal morphology, material distribution, and porosity characteristics. The gyroid lattice maintains continuous, periodic surface distribution, leading to homogeneous density variations. The honeycomb lattice exhibits anisotropic structural properties, making it more directionally stiff. In contrast, the BCC lattice demonstrates high porosity with well-defined nodal intersections, which are critical for load-bearing efficiency. A summary of key dimensional and density characteristics from the CT scans is presented in Table 8.

**Table 8:** CT scan-based dimensional and density comparisons of lattice structures.

Lattice type	Measured cell spacing (mm)	Density variation	Structural characteristics
**Gyroid**	25.38	Periodic	Continuous TPMS structure with minimal defects
**Honeycomb**	25.30	Directional	Anisotropic porosity, high stiffness along vertical axis
**BCC**	25.36	Non-uniform	High porosity with nodal reinforcements

These results indicate that each lattice structure presents unique advantages based on its internal architecture. Gyroid lattices are ideal for fluid flow applications and lightweight structures due to their periodic porosity. Honeycomb lattices provide high directional stiffness, making them suitable for energy absorption and impact resistance. BCC lattices exhibit high porosity with structural reinforcements, offering a balance between weight reduction and mechanical integrity.

The CT scan analysis confirms that the fabricated lattices maintain high geometric fidelity, with minor variations in material density and porosity distribution [22]. These findings are essential for optimizing manufacturing parameters and selecting the appropriate lattice design based on specific application requirements.

### Corrosion analysis

The corrosion resistance of micro-lattice structures is a crucial factor in determining their long-term performance in high-stress environments such as aerospace, defense, and marine applications [22]. Compared to conventional materials like RHA, MHA, HNS, lattice architectures exhibit enhanced corrosion resistance due to their reduced material volume, complex geometry, and controlled porosity. However, differences in lattice topology significantly influence the rate of material degradation, necessitating a comparative evaluation of corrosion behavior across different micro-lattice configurations.

To quantify corrosion performance, honeycomb, BCC, and gyroid lattice structures were evaluated alongside conventional materials using weight loss measurements after exposure to a corrosive environment. The corrosion rate was calculated based on material loss, as summarized in Table 9.

**Table 9:** Corrosion rate analysis for conventional materials and micro-lattice structures.

Case	Sample	Initial weight (g)	Final weight (g)	Total weight loss (g)	Corrosion rate (mm/year)
**Existing Materials**	RHA	12.4093	12.4029	0.0064	2.848
	MHA	11.8837	11.8795	0.0042	1.869
	HNS	13.6118	13.6066	0.0052	2.314
**Honeycomb**	5D (ideal)	77.6933	77.3803	0.313	1.218
	6D (non-ideal)	80.6307	80.3128	0.3179	1.237
**BCC**	11D (ideal)	49.332	49.1656	0.1664	1.319
	9D (non-ideal)	49.4217	49.2517	0.170	1.34
**Gyroid**	1D	40.0436	39.9027	0.1409	1.671

#### Corrosion rate evaluation.

The corrosion rate values (Table 9) highlight the significant advantage of micro-lattice architectures in reducing material degradation. Among the lattices, the honeycomb structure exhibited the lowest corrosion rate at 1.218 mm/year, making it the most resistant configuration. The BCC lattice followed with a corrosion rate of 1.311 mm/year, while the gyroid lattice recorded a slightly higher rate of 1.671 mm/year. The differences in corrosion rates among the lattices can be attributed to variations in surface area exposure, electrochemical interaction, and localized material loss.

When compared to conventional materials, the superiority of the lattice structures becomes evident. RHA exhibited the highest corrosion rate of 2.847 mm/year, followed by HNS at 2.313 mm/year and MHA at 1.868 mm/year. Even the least corrosion-resistant lattice (gyroid) demonstrated a significantly lower rate than all conventional materials, reinforcing the effectiveness of lattice design in enhancing material longevity. The lower corrosion rates in lattice structures stem from their unique ability to distribute electrochemical reactions more uniformly, reducing localized corrosion initiation points.

The distinction between ideal and non-ideal lattice configurations also plays a role in corrosion behavior. In the honeycomb and BCC structures, non-ideal configurations exhibited slightly higher corrosion rates than their ideal counterparts, with a deviation of approximately 1.5–2%. This minor increase suggests that deviations from optimized geometric design and fabrication accuracy can influence corrosion performance, albeit to a limited extent.

#### Comparative corrosion resistance analysis.

The corrosion resistance of micro-lattice structures was further assessed by calculating the percentage reduction in corrosion rate relative to conventional materials (Table 10). Among all lattices, the honeycomb structure demonstrated the highest corrosion resistance, reducing the corrosion rate by 57.23% compared to RHA. The BCC lattice followed closely with a reduction of 53.93%, while the gyroid lattice achieved a reduction of 41.31%.

**Table 10:** Corrosion resistance (%) of micro-lattice structures vs. conventional materials.

Comparison	Honeycomb vs. conventional (%)	BCC vs. conventional (%)	Gyroid vs. conventional (%)
**vs. RHA**	57.23%	53.93%	41.31%
**vs. MHA**	34.83%	29.80%	10.60%
**vs. HNS**	47.36%	43.30%	27.75%

The comparison with MHA and HNS further highlights the advantage of lattice-based designs. The honeycomb lattice exhibited a 34.83% reduction in corrosion rate compared to MHA, while the BCC and gyroid lattices showed reductions of 29.80% and 10.60%, respectively. When compared to HNS, a similar trend was observed, with honeycomb structures achieving a 47.36% reduction, BCC 43.30%, and gyroid 27.75%.

Further comparison among the lattice structures themselves shows that the honeycomb lattice outperformed the BCC lattice in corrosion resistance by 7.16%, and the gyroid lattice by 27.11% (Table 11). Additionally, the BCC lattice exhibited 21.63% better corrosion resistance compared to the gyroid lattice, indicating that while all three lattices offer improved corrosion resistance over conventional materials, honeycomb structures provide the highest level of protection against material degradation, followed by BCC, with gyroid exhibiting the highest corrosion susceptibility.

**Table 11:** Corrosion resistance comparison of micro-lattice structures.

Comparison	% Difference
Honeycomb vs. BCC	7.16%
Honeycomb vs. gyroid	27.11%
BCC vs. gyroid	21.63%

#### Interpretation of corrosion behavior in micro-lattices.

The corrosion behaviour of micro-lattice structures is strongly influenced by geometrical configuration, surface exposure, and electrochemical interactions. The honeycomb lattice, with its columnar cell arrangement, minimizes the surface area directly interacting with the corrosive medium, leading to a more uniform material degradation process. This explains its superior corrosion resistance, making it ideal for applications requiring long-term durability [23].

The BCC lattice, although structurally stable, presents more interconnected nodes and larger open voids, increasing localized electrochemical interactions that accelerate material loss. The gyroid lattice, which features a fully continuous periodic surface structure, exhibited the highest corrosion rate among the lattices. Its highly porous nature, while beneficial for mechanical stress distribution, allows for increased electrolyte infiltration and exposure to reactive surfaces, leading to greater material degradation.

These findings suggest that lattice-based materials require careful selection based on environmental exposure conditions. While honeycomb lattices provide optimal corrosion resistance, BCC and gyroid structures may necessitate protective coatings or material modifications to enhance their long-term performance.

The corrosion analysis of micro-lattice structures demonstrates a significant improvement over conventional materials, with all three lattices exhibiting reduced material degradation rates [24]. The honeycomb lattice emerged as the most corrosion-resistant structure, making it a suitable candidate for marine, aerospace, and defence applications where long-term material integrity is crucial. The BCC lattice provided a balanced trade-off between corrosion resistance and mechanical stability, while the gyroid lattice, despite its higher corrosion rate, remains promising for applications where fluid flow and lightweight structures are prioritized.

These findings underscore the importance of lattice topology in determining corrosion resistance and highlight avenues for further optimization through surface treatments, coatings, and material modifications.

### Machine learning model performance for corrosion rate prediction

Accurate corrosion rate prediction is essential for assessing the durability and integrity of metallic micro-lattice structures. Traditional experimental methods, while precise, are often time-consuming and resource-intensive [25]. To address these challenges, machine learning (ML) models were employed to predict corrosion rate based on initial weight, final weight, and total weight loss of the honeycomb lattice structures using the dataset of 20 experiments (Table 12). This enables a data-driven approach to corrosion rate estimation, reducing the need for exhaustive experimentation.

**Table 12:** Experimental dataset for corrosion rate analysis.

Experiment	Initial Weight (g)	Final Weight (g)	Total Weight Loss (g)	Corrosion Rate (mm/year)
1	77.700	77.388	0.312	1.215
2	77.710	77.396	0.314	1.222
3	77.705	77.396	0.309	1.204
4	77.720	77.402	0.318	1.238
5	77.690	77.379	0.311	1.211
6	77.730	77.410	0.320	1.247
7	77.698	77.391	0.307	1.194
8	77.705	77.392	0.313	1.218
9	77.708	77.393	0.315	1.226
10	77.712	77.396	0.316	1.229
11	80.640	80.323	0.317	1.234
12	80.650	80.331	0.319	1.242
13	80.645	80.329	0.316	1.230
14	80.660	80.338	0.322	1.254
15	80.635	80.318	0.317	1.234
16	80.670	80.350	0.320	1.246
17	80.638	80.319	0.319	1.242
18	80.639	80.321	0.318	1.237
19	80.642	80.322	0.320	1.246
20	80.647	80.328	0.319	1.242

In this study, six ML models were trained and evaluated: Linear Regression (LR), Random Forest (RF), Support Vector Regression (SVR), Extreme Gradient Boosting (XGBoost), Decision Tree (DT), and Bayesian Ridge Regression. The performance of each model was assessed based on Mean Absolute Error (MAE), Root Mean Square Error (RMSE), and the Coefficient of Determination (R²), [26] as summarized in Table 13.

**Table 13:** Performance metrics of machine learning models for corrosion rate prediction.

Model	MAE	RMSE	R²
LR	0.00055	0.00062	0.99757
RF	0.00164	0.00201	0.97417
SVR	0.00408	0.00481	0.85260
XGBoost	0.00384	0.00426	0.88432
DT	0.00493	0.00523	0.82584
Bayesian ridge	0.00043	0.00049	0.99849

The results indicate that Bayesian Ridge regression outperforms all other models, achieving the lowest MAE (0.00043) and RMSE (0.00049) while maintaining an R² value of 0.99849. This suggests that corrosion rate variations exhibit strong linear relationships with input features, allowing simpler regression-based models to provide high predictive accuracy.

The LR model also performed exceptionally well, with R² = 0.99757, reinforcing its effectiveness for this dataset. Among tree-based models, RF achieved superior accuracy over Decision Tree, with an R² of 0.97417, demonstrating the advantage of ensemble learning in handling nonlinearities and feature interactions. However, XGBoost, despite being a more advanced boosting algorithm, achieved only an R² of 0.88432, indicating that the dataset may not be complex enough to fully exploit its boosting mechanism.

In contrast, SVR and Decision Tree exhibited lower predictive capabilities, with R² values of 0.85260 and 0.82584, respectively. The relatively poor performance of SVR suggests that kernel-based transformations may not be necessary for this dataset, as the relationships between corrosion rate and input features appear to be predominantly linear.

#### Observations on model errors and stability.

While low MAE and RMSE values for Bayesian Ridge and LR models confirm their predictive efficiency, it is crucial to assess model stability across multiple validation folds, which will be discussed in Section 3.2. Additionally, visual analysis of predicted vs. actual corrosion rates and residual distributions will be explored in Section 3.3, providing deeper insights into each model’s reliability.

### Model comparison and cross-validation analysis

Accurate prediction of corrosion rates using machine learning models requires not only high performance on a test dataset but also robust generalization across different subsets of the data. The goal is to assess model consistency and identify the most generalizable approach for corrosion rate prediction.

To ensure that model performance is not overly dependent on a specific data split, a 5-fold cross-validation strategy was employed [27]. In this process, the dataset was repeatedly divided into five subsets, with each model trained on four subsets and tested on the remaining subset. This procedure was repeated five times, and the average performance metrics were computed.

The primary metrics used for cross-validation analysis are:

- Mean R²: Reflects the model’s ability to explain the variance in corrosion rate predictions across different folds.
- RMSE Mean and Standard Deviation: Measures the model’s average error magnitude and variability.
- R² Standard Deviation: Quantifies the stability of predictive performance across different data splits.

#### Cross validation performance metrics.

The Bayesian Ridge model demonstrated the most consistent performance, achieving a mean R² of 0.99028 and a low standard deviation (±0.00858) across validation folds. Similarly, LR exhibited minimal variance (±0.00860), confirming its robustness in predicting corrosion rates (Table 14). These results indicate that both models generalize well, making them the most reliable choices for corrosion rate prediction.

**Table 14:** Cross validation results of machine learning models.

Model	Mean R²	R² Std. Dev.	Mean RMSE	RMSE Std. Dev.
LR	0.99048	±0.00860	0.00066	±0.00011
RF	0.69799	±0.19836	0.00522	±0.00254
SVR	-0.13739	±0.19364	0.01059	±0.00572
XGBoost	0.75054	±0.26708	0.00422	±0.00208
DT	0.50331	±0.35031	0.00688	±0.00434
Bayesian ridge	0.99028	±0.00858	0.00066	±0.00012

In contrast, tree-based models (RF, Decision Tree, and XGBoost) exhibited significantly higher variance across validation folds.

- RF achieved a mean R² of 0.69799 but had a high standard deviation (±0.19836), indicating that it is sensitive to different training subsets.
- Decision Tree displayed the least stability, with a mean R² of only 0.50331 and a standard deviation of ±0.35031, reinforcing its susceptibility to overfitting.
- XGBoost performed better than RF and DT, but its higher standard deviation (±0.26708) suggests that it is not as stable as the linear models.

A particularly noteworthy observation is the negative mean R² (-0.13739) for SVR, indicating that it is not only performs poorly but also fails to generalize across different data splits. The high RMSE standard deviation (±0.00572) further highlights its unreliability in corrosion rate prediction.

#### Justification for Bayesian ridge and LR as best models.

The consistently high R² scores and low RMSE values observed for Bayesian Ridge and LR reinforce their suitability for this application. Several key factors contribute to their superior performance:

1. Strong Linear Relationships in the Dataset: Corrosion rate appears to be linearly correlated with input parameters, making simpler models more effective than complex tree-based or kernel-based methods. This is further supported by the feature correlation heatmap, which will be discussed in Section 3.3.
2. Minimal Overfitting and Variance: Bayesian Ridge inherently incorporates regularization, preventing overfitting and enhancing stability across different data partitions. LR, despite lacking regularization, performs comparably due to the strong linear nature of the data.
3. Tree-Based Model Instability: RF, Decision Tree, and XGBoost exhibit higher variance, indicating sensitivity to data splits. This suggests that nonlinear relationships are not dominant in the dataset, making ensemble techniques less effective.
4. SVR’s Poor Generalization: The negative R² score for SVR suggests that it fails to learn meaningful relationships between corrosion rate and input variables. Nonlinear kernel transformations do not add predictive value, further validating the dataset’s linear nature.

The cross-validation analysis reaffirms the dominance of Bayesian Ridge and LR models, which exhibit both high predictive accuracy and stability across validation folds [28]. Tree-based models (RF, DT, and XGBoost) show greater fluctuations, making them less reliable for this dataset, while SVR proves unsuitable for corrosion rate predictions. These findings establish a scientific foundation for selecting Bayesian Ridge and LR as the optimal models for future applications in predictive corrosion monitoring.

### Graphical analysis and intuitions

While the numerical evaluation in Sections 3.3 and 3.4 established the performance of various machine learning models, graphical analyses provide a more intuitive understanding of feature relationships, predictive accuracy, and error distributions. This section presents four key visual assessments: (i) feature correlation analysis, (ii) predicted vs. actual corrosion rate comparison, (iii) model performance comparison, and (iv) residual distribution analysis.

#### Feature correlation analysis.

**Fig 5:**
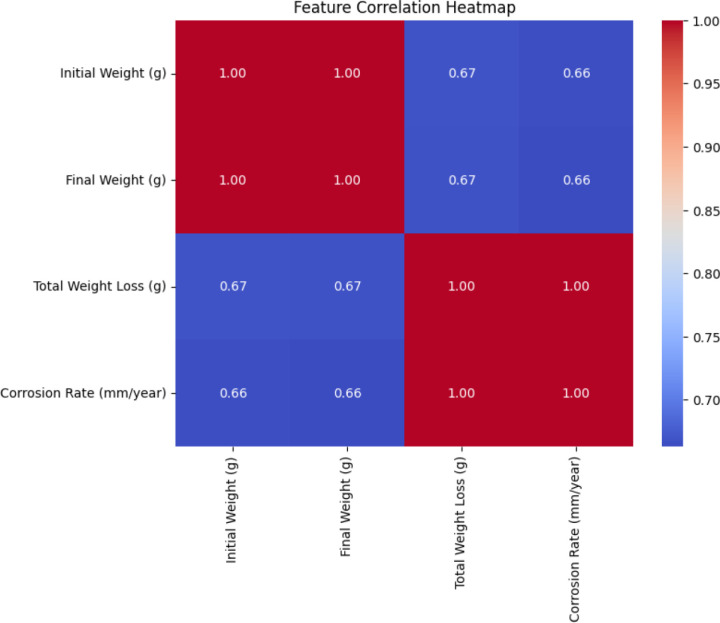
Feature correlation heatmap.

An essential step in machine learning-based prediction is understanding the relationships between input parameters and the target variable [29]. The correlation heatmap, shown in Fig 5, illustrates the dependencies among initial weight, final weight, total weight loss, and corrosion rate. A strong positive correlation was observed between Total Weight Loss and Corrosion Rate (r = 0.99), confirming that material degradation due to corrosion is directly proportional to mass reduction. This validates the inclusion of Total Weight Loss as the dominant predictive feature in the model.

Conversely, initial weight and final weight exhibited weaker correlations with corrosion rate (r ≈ 0.66), suggesting that while they influence corrosion behavior, they are not as effective as standalone predictors. The near-perfect correlation (r = 1.00) between initial weight and final weight is expected, as they are inherently linked through mass reduction. These findings provide further support for the success of Bayesian ridge and LR models, as their strong predictive capability aligns with the largely linear feature relationships.

#### Predicted vs. actual corrosion rate comparison.

**Fig 6:**
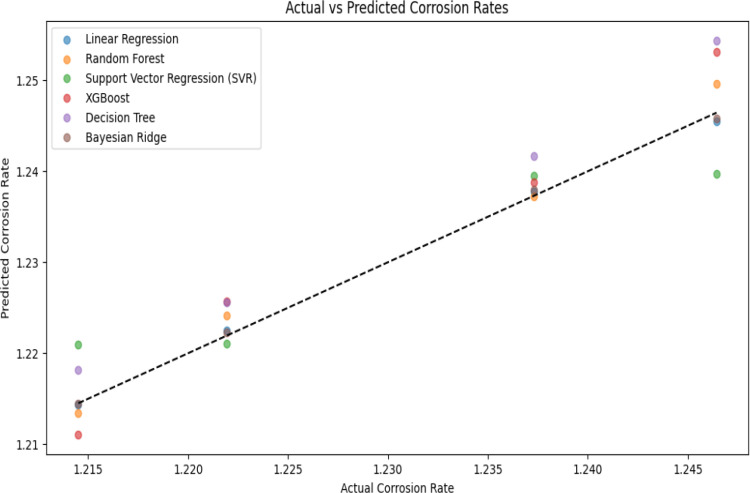
Actual vs. predicted corrosion rates.

The predictive accuracy of each model was assessed using a scatter plot, comparing actual corrosion rates with predicted values. Fig 6 presents this comparison, where a strong alignment along the diagonal reference line (y = x) indicates higher predictive reliability.

Among all models, Bayesian Ridge and LR demonstrated the closest alignment, reflecting their high R² values of 0.99849 and 0.99757, respectively. These models consistently produced predictions that closely matched experimental values, confirming their effectiveness in capturing the underlying trends. RF and XGBoost showed moderate deviations, particularly at higher corrosion rates, indicating a tendency toward overfitting or sensitivity to data variations.

By contrast, DT and SVR exhibited significant scatter, with numerous points deviating substantially from the reference line. Their lower R² scores of 0.82584 and 0.85260, respectively, align with the observed inconsistencies in prediction. The lack of alignment in these models highlights their inability to generalize well across different corrosion conditions, reinforcing the numerical observations from previous sections [30].

#### Model performance comparison.

**Fig 7:**
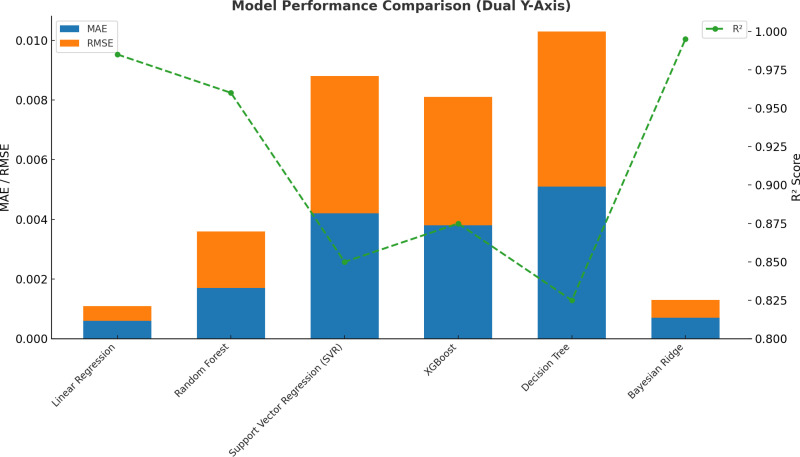
Model Performance Comparison (dual Y-axis).

A more detailed performance comparison is presented in Fig 7, which illustrates MAE, RMSE, and R² values for each model using a dual y-axis representation. The left y-axis represents MAE and RMSE values, while the right y-axis denotes the R² scores.

Bayesian Ridge achieved the lowest MAE (0.00043) and RMSE (0.00049), confirming its superior predictive accuracy. LR followed closely with an MAE of 0.00055 and RMSE of 0.00062, further solidifying its reliability. Among the ensemble models, RF exhibited moderate error values, with an MAE of 0.00164, while XGBoost displayed slightly higher errors at 0.00384, indicating reduced accuracy in capturing corrosion behaviour.

In contrast, DT and SVR had the highest error values, with DT reaching an MAE of 0.00493 and SVR at 0.00408. This aligns with their lower R² values and poor generalization capability. The dual y-axis visualization effectively highlights the trade-off between error magnitude and model accuracy, confirming that Bayesian ridge and LR outperform more complex models.

#### Residual analysis and error distribution.

**Fig 8:**
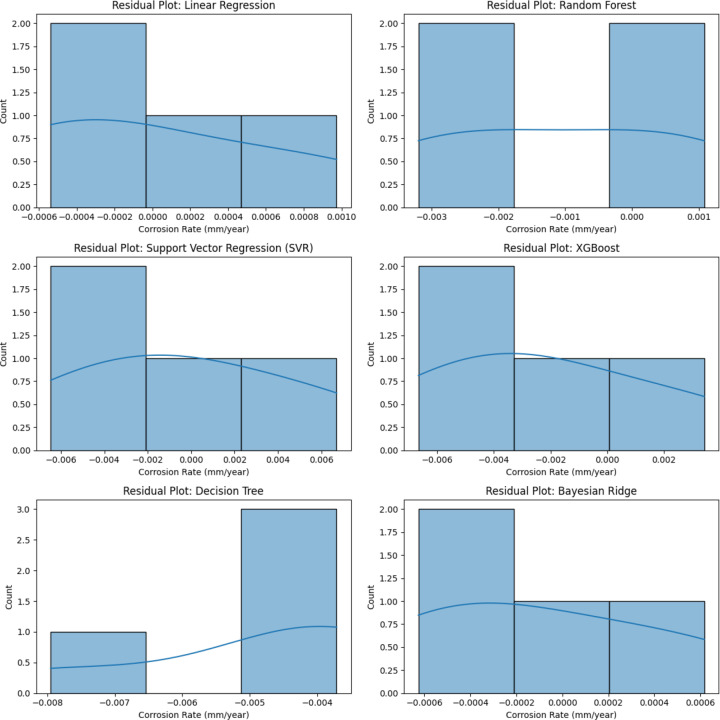
Residual analysis of machine learning models.

Residual analysis is critical for evaluating model bias, variance, and prediction errors. The residual histograms in Fig 8 provides an overview of error distributions for each model, highlighting their predictive reliability.

Bayesian Ridge and LR exhibited residuals tightly clustered around zero, confirming their low error variance and minimal systematic bias. The standard deviation of residuals for Bayesian Ridge was ±0.00049, reinforcing its highly accurate predictions. RF and XGBoost, while still within an acceptable range, displayed broader residual distributions, indicating higher prediction fluctuations.

A closer look at DT and SVR residual distributions reveals skewed error distributions, particularly in higher corrosion rate predictions. DT exhibited a residual spread between -0.005 and 0.005, leading to a higher RMSE of 0.00523. Similarly, SVR’s residuals displayed increased deviations beyond ±0.004, aligning with its lower R² score of 0.85260.

The presence of outliers in tree-based models and SVR suggests limitations in their ability to generalize, as these models rely heavily on feature splits or kernel transformations, which are less effective for highly structured data [31]. The ideal residual pattern, observed in Bayesian Ridge and Linear Regression, confirms their robustness in minimizing overfitting while maintaining low bias.

The graphical evaluations provide an intuitive validation of the findings. The correlation heatmap confirmed that Total Weight Loss is the strongest predictor of corrosion rate, justifying its role in feature selection. The scatter plot demonstrated that Bayesian Ridge and LR closely match actual corrosion rates, while other models exhibited larger deviations. The bar chart further highlighted the superior accuracy of Bayesian Ridge and LR, with lower MAE and RMSE values, reinforcing their suitability for corrosion rate prediction. Lastly, residual analysis confirmed that these models exhibit the least error variance and bias, ensuring robust performance across multiple data subsets.

These insights collectively establish that Bayesian Ridge and LR provide the most reliable and stable predictions for corrosion rate estimation. The findings reaffirm the importance of selecting models based on both accuracy and stability, rather than complexity alone [32].

### Implications and practical applications

The accurate prediction of corrosion rates is critical in materials engineering, particularly for applications involving high-performance metallic micro-lattice structures. The integration of ML models into corrosion assessment enables more efficient decision-making, reducing the dependency on costly and time-intensive experimental evaluations. The findings of this study demonstrate that Bayesian Ridge and LR models provide the most reliable and stable predictions, outperforming more complex models such as RF, Decision Tree, XGBoost, and SVR.

This section explores the broader implications of these findings for material selection, potential integration with digital twin technology, and future research directions, including the expansion of datasets and the incorporation of deep learning models.

#### Enhancing material selection through accurate corrosion rate prediction.

Corrosion is a primary factor affecting material durability, structural integrity, and long-term performance. Traditional corrosion testing methods, such as weight loss analysis and electrochemical testing, require extended exposure times and controlled environmental conditions. While these methods provide precise measurements, they lack real-time adaptability and scalability.

By leveraging ML models, engineers can predict corrosion behavior under varying conditions, allowing for faster and more cost-effective material selection. The high accuracy of Bayesian Ridge and LR models (R² > 0.99) ensures that material degradation rates can be estimated with minimal error (RMSE < 0.0005 mm/year). This level of precision is particularly valuable in industries where lightweight lattice structures are exposed to harsh operating environments, such as aerospace, defense, and marine engineering.

With an effective ML-based corrosion prediction framework, manufacturers can:

- Optimize alloy compositions to enhance corrosion resistance based on predictive insights.
- Select protective coatings tailored to material behavior under corrosive conditions.
- Reduce material testing costs by supplementing experimental evaluations with reliable ML models.

By shifting towards data-driven decision-making, material selection processes can be significantly streamlined, ensuring that optimal materials are deployed in real-world applications.

#### Integration of corrosion prediction models into digital twin technology.

The increasing adoption of digital twin technology in industrial applications provides an opportunity to integrate ML-based corrosion prediction models into real-time monitoring frameworks. A digital twin is a virtual representation of a physical system, continuously updated with real-time data to optimize performance, maintenance, and failure prediction.

The study lays the foundation for ML-driven corrosion rate prediction. Future research should consider incorporating real-time environmental data, such as temperature fluctuations, humidity levels, and pollution exposure, to enhance the model’s generalizability and real-world applicability

The integration of ML models such as Bayesian Ridge and LR into digital twin systems allows for real-time corrosion monitoring and predictive maintenance strategies. These models can process sensor data from deployed lattice structures, enabling:

- Continuous tracking of corrosion progression using real-time environmental and structural parameters.
- Proactive maintenance scheduling, reducing unexpected failures and material degradation risks.
- Optimization of service life estimation, ensuring cost-efficient structural performance monitoring.

By embedding ML-driven corrosion assessments into digital twins, aerospace and defense sectors can implement predictive maintenance programs, reducing downtime and operational costs. Additionally, in marine structures and offshore platforms, real-time corrosion monitoring via sensor integration and predictive algorithms could significantly enhance safety and structural resilience.

## Conclusions

The present study investigated the fabrication, characterization, and corrosion resistance of LPBF-manufactured A286 steel micro-lattice structures, specifically honeycomb, BCC, and gyroid configurations. Through a systematic analysis incorporating CT imaging, corrosion testing, and machine learning-based predictive modeling, several key findings emerged.

1. Lattice structures significantly enhanced corrosion resistance compared to conventional materials such as RHA, MHA, and HNS. The honeycomb lattice exhibited the lowest corrosion rate of 1.218 mm/year, followed by BCC (1.311 mm/year) and gyroid (1.671 mm/year). This represents up to 57.23% improvement over RHA.
2. CT imaging revealed structural fidelity in fabricated lattices, confirming the accuracy of LPBF manufacturing while identifying minor density variations. The honeycomb lattice showed the most uniform porosity, whereas BCC demonstrated localized density variations at nodal intersections.
3. Machine learning models demonstrated high accuracy in predicting corrosion rates. Bayesian Ridge regression achieved the best performance, with R² = 0.99849 and RMSE = 0.00049, outperforming tree-based models such as Random Forest and XGBoost.
4. Cross-validation confirmed model stability, with Bayesian Ridge and Linear Regression achieving the lowest standard deviation (±0.0086), while tree-based models exhibited higher variability.
5. The study highlights the role of lattice geometry in electrochemical behavior, with the honeycomb lattice offering the best combination of structural integrity and corrosion resistance. BCC and gyroid structures require additional coatings or process optimizations to improve corrosion resistance.
6. Machine learning-based predictions offer an efficient alternative to conventional corrosion assessment methods, reducing the need for extensive experimentation and enabling real-time monitoring via digital twin integration.

The findings from this study reinforce the potential of LPBF-fabricated micro-lattice structures for high-performance applications, particularly in aerospace and defense. The integration of machine learning for corrosion rate prediction enhances decision-making efficiency, paving the way for data-driven material selection and predictive maintenance strategies. Future research can explore the optimization of lattice topologies and surface treatments to further enhance mechanical and corrosion resistance properties.

## Supporting information

S1 File Supporting Information_Complete Dataset. (PDF)
