# Supplementary material for: Performance evaluation of machine learning techniques in surface morphology and corrosion prediction for A286 3D printed micro-lattice structures
Source: PLoS One. 2025 May 7;20(5):e0320565. doi: 10.1371/journal.pone.0320565 (PMC12058171; doi:10.1371/journal.pone.0320565)
Supplement: S1 File — (PDF) [file pone.0320565.s001.pdf]

Supporting Information_Complete Dataset

```
import pandas as pd
from sklearn.model_selection import train_test_split
from sklearn.linear_model import LinearRegression
from sklearn.ensemble import RandomForestRegressor
from sklearn.metrics import mean_absolute_error, mean_squared_error,
r2_score

# Load the dataset
data = {
    "Initial Weight (g)": [77.700, 77.710, 77.705, 77.720, 77.690,
77.730, 77.698, 77.705, 77.708, 77.712,
                            80.640, 80.650, 80.645, 80.660, 80.635,
80.670, 80.638, 80.639, 80.642, 80.647],
    "Final Weight (g)": [77.388, 77.396, 77.396, 77.402, 77.379,
77.410, 77.391, 77.392, 77.393, 77.396,
                          80.323, 80.331, 80.329, 80.338, 80.318,
80.350, 80.319, 80.321, 80.322, 80.328],
    "Total Weight Loss (g)": [0.312, 0.314, 0.309, 0.318, 0.311,
0.320, 0.307, 0.313, 0.315, 0.316,
                               0.317, 0.319, 0.316, 0.322, 0.317,
0.320, 0.319, 0.318, 0.320, 0.319],
    "Corrosion Rate (mm/year)": [1.21451, 1.22193, 1.20377, 1.23803,
1.21092, 1.24663, 1.19436, 1.21822,
                                  1.22564, 1.22933, 1.23392, 1.24163,
1.22959, 1.25439, 1.23392, 1.24644,
                                  1.24163, 1.23730, 1.24644, 1.24163]
}

df = pd.DataFrame(data)

# Features and target
X = df[["Initial Weight (g)", "Final Weight (g)", "Total Weight Loss
(g)"]]
y = df["Corrosion Rate (mm/year)"]

# Train-test split
X_train, X_test, y_train, y_test = train_test_split(X, y,
test_size=0.2, random_state=42)

# Linear Regression Model
lr_model = LinearRegression()
lr_model.fit(X_train, y_train)
lr_predictions = lr_model.predict(X_test)

# Random Forest Regressor Model
rf_model = RandomForestRegressor(random_state=42, n_estimators=100,
max_depth=5)
```

```
rf_model.fit(X_train, y_train)
rf_predictions = rf_model.predict(X_test)

# Evaluation function
def evaluate_model(name, y_true, y_pred):
    mae = mean_absolute_error(y_true, y_pred)
    mse = mean_squared_error(y_true, y_pred)
    rmse = mse ** 0.5
    r2 = r2_score(y_true, y_pred)
    print(f"{name} Model Evaluation:")
    print(f"  MAE: {mae:.5f}")
    print(f"  MSE: {mse:.5f}")
    print(f"  RMSE: {rmse:.5f}")
    print(f"  R²: {r2:.5f}\n")

# Evaluate both models
evaluate_model("Linear Regression", y_test, lr_predictions)
evaluate_model("Random Forest", y_test, rf_predictions)
```

```
Linear Regression Model Evaluation:
  MAE: 0.00055
  MSE: 0.00000
  RMSE: 0.00062
  R²: 0.99757

Random Forest Model Evaluation:
  MAE: 0.00164
  MSE: 0.00000
  RMSE: 0.00201
  R²: 0.97417
```

```
from sklearn.model_selection import cross_val_score
import numpy as np

# Define RMSE scoring function
def rmse_scorer(estimator, X, y):
    from sklearn.metrics import mean_squared_error
    y_pred = estimator.predict(X)
    return np.sqrt(mean_squared_error(y, y_pred))

# Cross-validation for Linear Regression
lr_cv_scores = cross_val_score(lr_model, X, y, cv=5, scoring='r2')  #
R² score
lr_rmse_cv = cross_val_score(lr_model, X, y, cv=5,
scoring=rmse_scorer)  # RMSE

print("Linear Regression Cross-Validation:")
print(f"  Mean R²: {np.mean(lr_cv_scores):.5f} (+/-
{np.std(lr_cv_scores):.5f})")
```

```
print(f"  Mean RMSE: {np.mean(lr_rmse_cv):.5f} (+/- {np.std(lr_rmse_cv):.5f})\\n")

# Cross-validation for Random Forest
rf_cv_scores = cross_val_score(rf_model, X, y, cv=5, scoring='r2')  # R² score
rf_rmse_cv = cross_val_score(rf_model, X, y, cv=5, scoring=rmse_scorer)  # RMSE

print("Random Forest Cross-Validation:")
print(f"  Mean R²: {np.mean(rf_cv_scores):.5f} (+/- {np.std(rf_cv_scores):.5f})")
print(f"  Mean RMSE: {np.mean(rf_rmse_cv):.5f} (+/- {np.std(rf_rmse_cv):.5f})")
```

```
Linear Regression Cross-Validation:
  Mean R²: 0.99048 (+/- 0.00860)
  Mean RMSE: 0.00066 (+/- 0.00011)\n
Random Forest Cross-Validation:
  Mean R²: 0.69799 (+/- 0.19836)
  Mean RMSE: 0.00522 (+/- 0.00254)
```

```
import matplotlib.pyplot as plt
import numpy as np

# Predicted vs Actual for Linear Regression
plt.figure(figsize=(12, 6))
plt.subplot(1, 2, 1)
plt.scatter(y_test, lr_predictions, color='blue', label='Linear Regression', alpha=0.7)
plt.plot([y.min(), y.max()], [y.min(), y.max()], 'k--', lw=2, label='Ideal Fit')
plt.title('Predicted vs Actual (Linear Regression)')
plt.xlabel('Actual Corrosion Rate')
plt.ylabel('Predicted Corrosion Rate')
plt.legend()
plt.grid()

# Predicted vs Actual for Random Forest
plt.subplot(1, 2, 2)
plt.scatter(y_test, rf_predictions, color='green', label='Random Forest', alpha=0.7)
plt.plot([y.min(), y.max()], [y.min(), y.max()], 'k--', lw=2, label='Ideal Fit')
plt.title('Predicted vs Actual (Random Forest)')
plt.xlabel('Actual Corrosion Rate')
plt.ylabel('Predicted Corrosion Rate')
plt.legend()
plt.grid()
```

```
plt.tight_layout()
plt.show()


# Residual Plot for Linear Regression
plt.figure(figsize=(6, 6))
lr_residuals = y_test - lr_predictions
plt.scatter(lr_predictions, lr_residuals, color='blue', alpha=0.7)
plt.axhline(0, color='red', linestyle='--', lw=2)
plt.title('Residual Plot (Linear Regression)')
plt.xlabel('Predicted Corrosion Rate')
plt.ylabel('Residuals')
plt.grid()
plt.show()

# Cross-Validation Scores Bar Chart
models = ['Linear Regression', 'Random Forest']
r2_scores = [np.mean(lr_cv_scores), np.mean(rf_cv_scores)]
r2_std = [np.std(lr_cv_scores), np.std(rf_cv_scores)]

plt.figure(figsize=(8, 6))
plt.bar(models, r2_scores, yerr=r2_std, color=['blue', 'green'],
alpha=0.7, capsize=10)
plt.title('Cross-Validation R² Scores')
plt.ylabel('Mean R²')
plt.grid(axis='y')
plt.show()
```

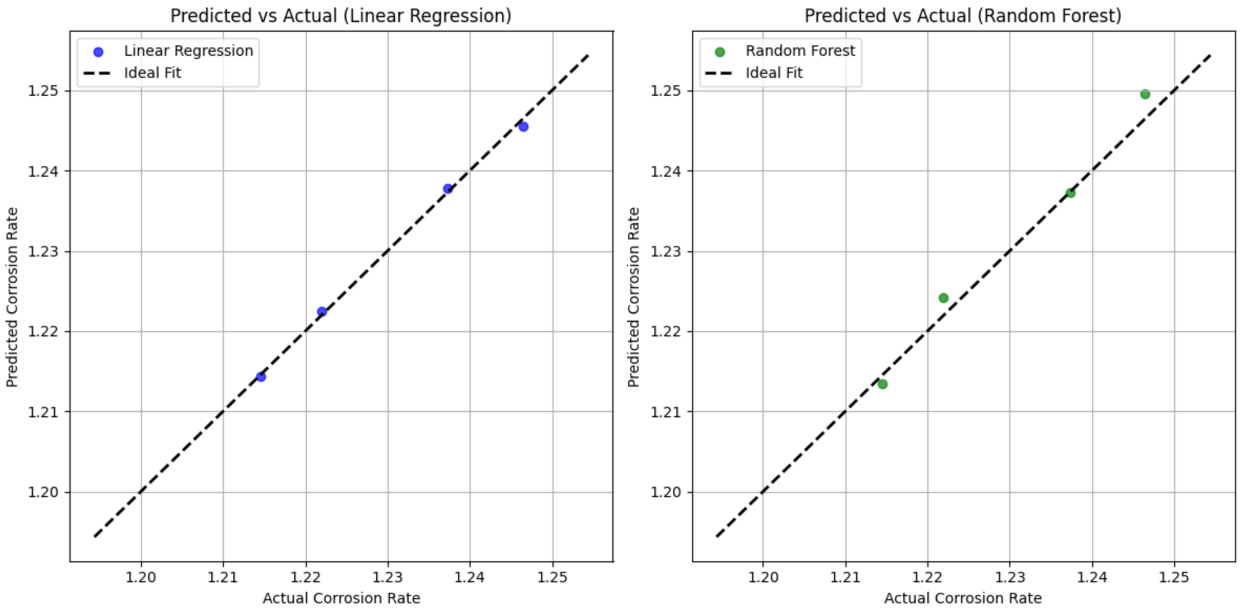

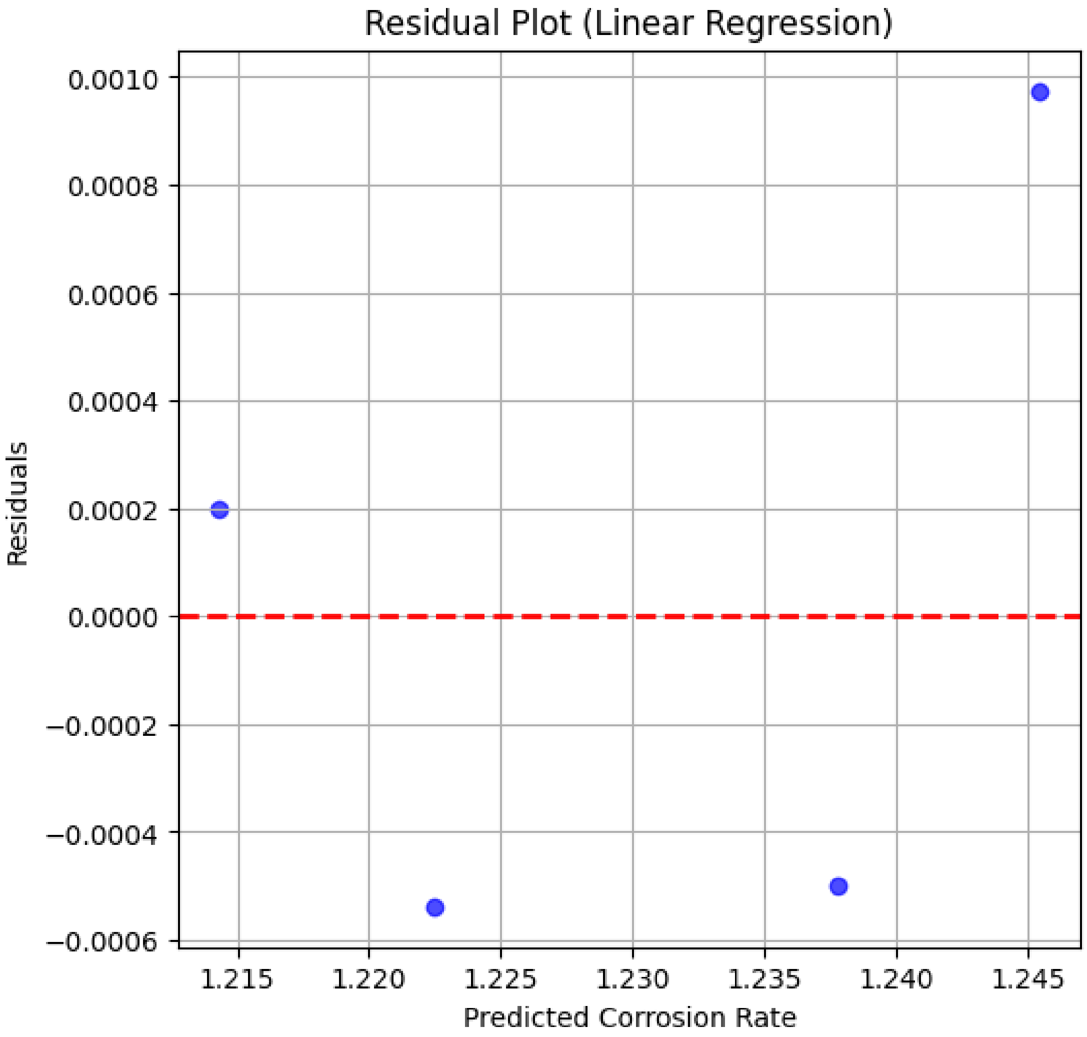

Residual Plot (Linear Regression)
Residuals
0.0010
0.0008
0.0006
0.0004
0.0002
0.0000
−0.0002
−0.0004
−0.0006
1.215
1.220
1.225
1.230
1.235
1.240
1.245
Predicted Corrosion Rate

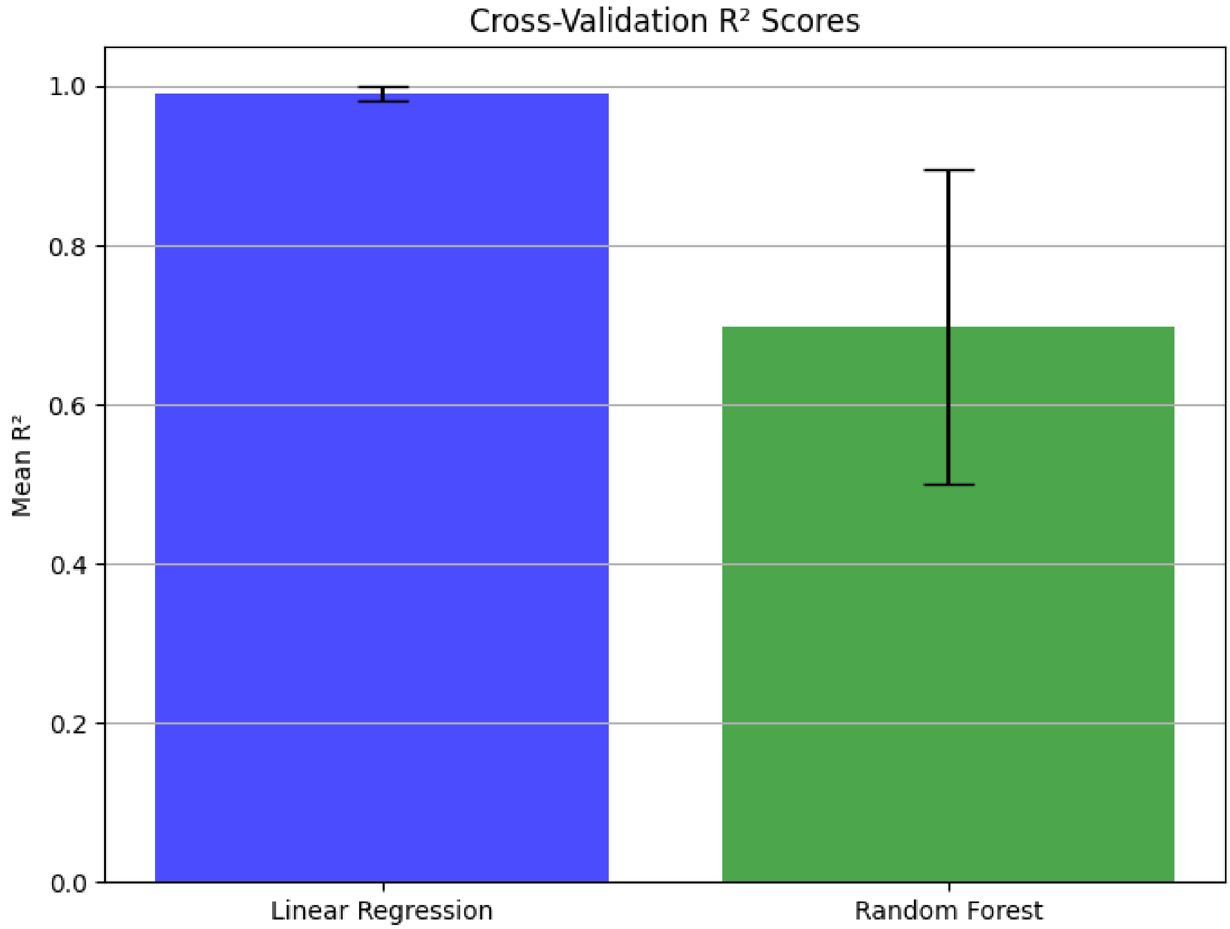


```
plt.savefig()

---------------------------------------------------------------------------
TypeError                                 Traceback (most recent call
last)
<ipython-input-5-a295b41f84dc> in <cell line: 1>()
----> 1 plt.savefig()

/usr/local/lib/python3.10/dist-packages/matplotlib/pyplot.py in
savefig(*args, **kwargs)
   1021 def savefig(*args, **kwargs):
   1022     fig = gcf()
-> 1023     res = fig.savefig(*args, **kwargs)
   1024     fig.canvas.draw_idle()  # Need this if 'transparent=True',
to reset colors.
   1025     return res

TypeError: Figure.savefig() missing 1 required positional argument:
'fname'
```

```
<Figure size 640x480 with 0 Axes>

# Importing necessary libraries
import pandas as pd
import numpy as np
from sklearn.model_selection import train_test_split, cross_val_score
from sklearn.tree import DecisionTreeRegressor
from sklearn.metrics import mean_absolute_error, mean_squared_error,
r2_score
import matplotlib.pyplot as plt

# Correcting the dataset to ensure all arrays have the same length (20
entries)
data = {
    'Initial Weight': [77.700, 77.710, 77.705, 77.720, 77.690, 77.730,
77.698, 77.705, 77.708, 77.712,
                       80.640, 80.650, 80.645, 80.660, 80.635, 80.670,
80.638, 80.639, 80.642, 80.647],
    'Final Weight': [77.388, 77.396, 77.396, 77.402, 77.379, 77.410,
77.391, 77.392, 77.393, 77.396,
                     80.323, 80.331, 80.329, 80.338, 80.318, 80.350,
80.319, 80.321, 80.322, 80.328],
    'Total Weight Loss': [0.312, 0.314, 0.309, 0.318, 0.311, 0.320,
0.307, 0.313, 0.315, 0.316,
                          0.317, 0.319, 0.316, 0.322, 0.317, 0.320,
0.319, 0.318, 0.320, 0.319],
    'Corrosion Rate': [1.21451, 1.22193, 1.20377, 1.23803, 1.21092,
1.24663, 1.19436, 1.21822, 1.22564, 1.22933,
                       1.23392, 1.24163, 1.22959, 1.25439, 1.23392,
1.24644, 1.24163, 1.23730, 1.24644, 1.24163]
}

# Convert the corrected data into a DataFrame
df = pd.DataFrame(data)

# Features (independent variables)
X = df[['Initial Weight', 'Final Weight', 'Total Weight Loss']]

# Target variable (dependent variable)
y = df['Corrosion Rate']

# Splitting the dataset into training and testing sets (80% training,
20% testing)
X_train, X_test, y_train, y_test = train_test_split(X, y,
test_size=0.2, random_state=42)

# Initialize the Decision Tree Regressor model
dt_model = DecisionTreeRegressor(random_state=42)

# Train the model
```

```
dt_model.fit(X_train, y_train)

# Predictions
y_pred = dt_model.predict(X_test)

# Evaluation Metrics
mae = mean_absolute_error(y_test, y_pred)
rmse = np.sqrt(mean_squared_error(y_test, y_pred))
r2 = r2_score(y_test, y_pred)

# Cross-validation score
cv_score = cross_val_score(dt_model, X, y, cv=5, scoring='r2')

# Display results
print(f'Mean Absolute Error (MAE): {mae}')
print(f'Root Mean Squared Error (RMSE): {rmse}')
print(f'R-squared (R²): {r2}')
print(f'Cross-validation R² scores: {cv_score}')
print(f'Mean Cross-validation R²: {cv_score.mean()}')

# Visualization: Predicted vs. Actual Corrosion Rates
plt.figure(figsize=(8, 6))
plt.scatter(y_test, y_pred, color='blue')
plt.plot([min(y_test), max(y_test)], [min(y_test), max(y_test)],
color='red', linestyle='--')
plt.xlabel('Actual Corrosion Rate')
plt.ylabel('Predicted Corrosion Rate')
plt.title('Predicted vs Actual Corrosion Rates (Decision Tree)')
plt.show()

Mean Absolute Error (MAE): 0.004925000000000013
Root Mean Squared Error (RMSE): 0.00523162498656009
R-squared (R²): 0.8258415053279378
Cross-validation R² scores: [-0.16214403  0.69694453  0.46547114
0.78942075  0.72686803]
Mean Cross-validation R²: 0.5033120855951486
```

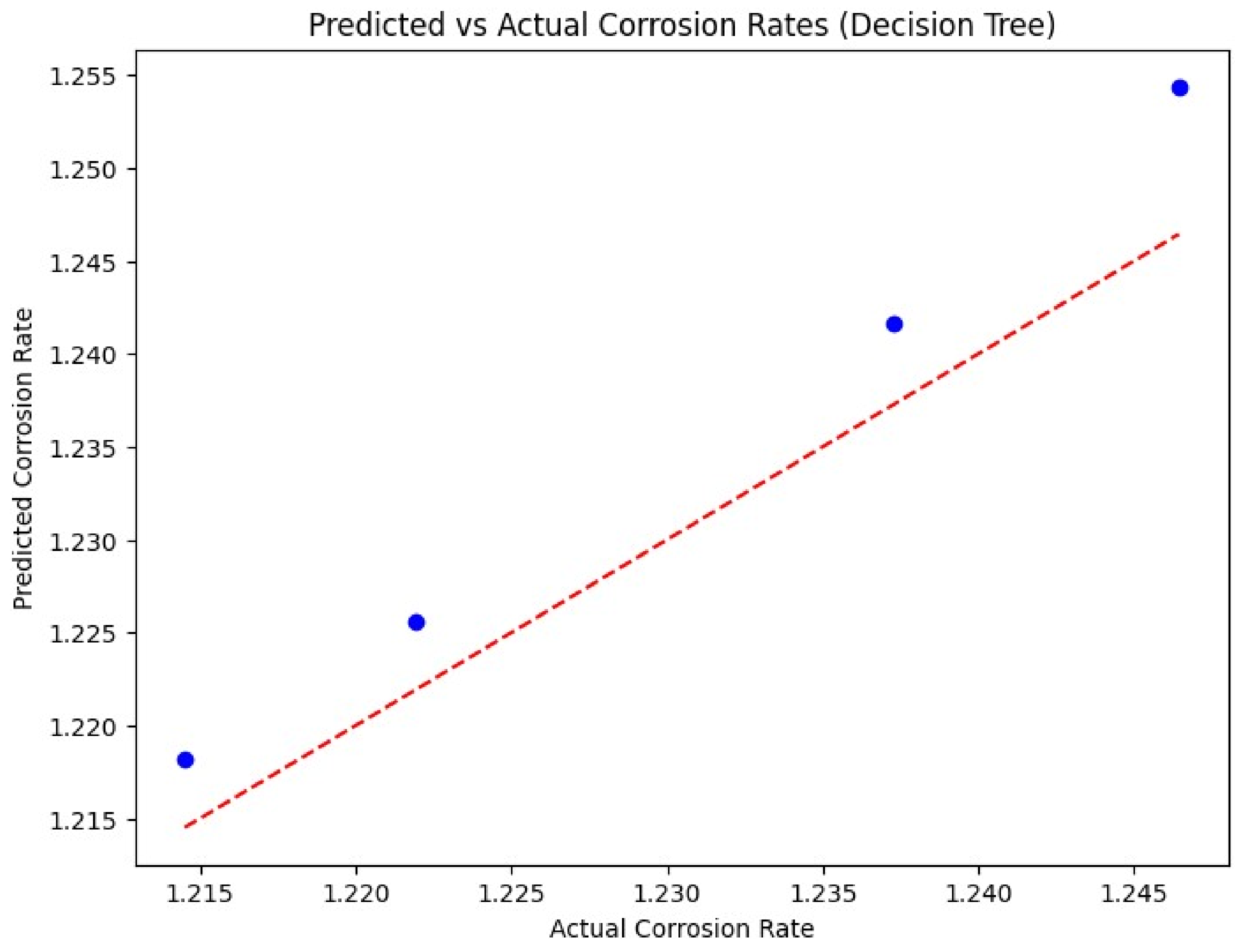


```
import pandas as pd
import numpy as np
from sklearn.model_selection import train_test_split, cross_val_score
from sklearn.linear_model import LinearRegression, BayesianRidge
from sklearn.ensemble import RandomForestRegressor
from sklearn.svm import SVR
from xgboost import XGBRegressor
from sklearn.tree import DecisionTreeRegressor
from sklearn.neural_network import MLPRegressor
from sklearn.metrics import mean_absolute_error, mean_squared_error,
r2_score

# Load the dataset
data = {
    "Initial Weight (g)": [77.700, 77.710, 77.705, 77.720, 77.690,
77.730, 77.698, 77.705, 77.708, 77.712,
                            80.640, 80.650, 80.645, 80.660, 80.635,
80.670, 80.638, 80.639, 80.642, 80.647],
    "Final Weight (g)": [77.388, 77.396, 77.396, 77.402, 77.379,
77.410, 77.391, 77.392, 77.393, 77.396,
```

```
                          80.323, 80.331, 80.329, 80.338, 80.318,
80.350, 80.319, 80.321, 80.322, 80.328],
    "Total Weight Loss (g)": [0.312, 0.314, 0.309, 0.318, 0.311,
0.320, 0.307, 0.313, 0.315, 0.316,
                               0.317, 0.319, 0.316, 0.322, 0.317,
0.320, 0.319, 0.318, 0.320, 0.319],
    "Corrosion Rate (mm/year)": [1.21451, 1.22193, 1.20377, 1.23803,
1.21092, 1.24663, 1.19436, 1.21822,
                                  1.22564, 1.22933, 1.23392, 1.24163,
1.22959, 1.25439, 1.23392, 1.24644,
                                  1.24163, 1.23730, 1.24644, 1.24163]
}

df = pd.DataFrame(data)

# Features and target
X = df[["Initial Weight (g)", "Final Weight (g)", "Total Weight Loss
(g)"]]
y = df["Corrosion Rate (mm/year)"]

# Train-test split
X_train, X_test, y_train, y_test = train_test_split(X, y,
test_size=0.2, random_state=42)

# Define models
models = {
    "Linear Regression": LinearRegression(),
    "Random Forest": RandomForestRegressor(random_state=42,
n_estimators=100, max_depth=5),
    "Support Vector Regression (SVR)": SVR(kernel="rbf", C=10,
epsilon=0.01),
    "XGBoost": XGBRegressor(objective="reg:squarederror",
n_estimators=100, learning_rate=0.1, max_depth=3),
    "Decision Tree": DecisionTreeRegressor(random_state=42,
max_depth=5),
    "Bayesian Ridge": BayesianRidge(),
    "Neural Network (ANN)": MLPRegressor(hidden_layer_sizes=(64, 32),
max_iter=1000, random_state=42)
}

# Function to evaluate models
def evaluate_model(name, model, X_train, X_test, y_train, y_test):
    model.fit(X_train, y_train)
    y_pred = model.predict(X_test)

    mae = mean_absolute_error(y_test, y_pred)
    mse = mean_squared_error(y_test, y_pred)
    rmse = mse ** 0.5
    r2 = r2_score(y_test, y_pred)
```

```
    print(f"{name} Model Evaluation:")
    print(f"  MAE: {mae:.5f}")
    print(f"  MSE: {mse:.5f}")
    print(f"  RMSE: {rmse:.5f}")
    print(f"  R²: {r2:.5f}\n")

    return model  # Return trained model for cross-validation

# Train and evaluate each model
trained_models = {}
for name, model in models.items():
    trained_models[name] = evaluate_model(name, model, X_train,
X_test, y_train, y_test)

# Cross-validation function
def rmse_scorer(estimator, X, y):
    y_pred = estimator.predict(X)
    return np.sqrt(mean_squared_error(y, y_pred))

# Perform cross-validation
print("\nCross-Validation Results:\n")
for name, model in trained_models.items():
    r2_scores = cross_val_score(model, X, y, cv=5, scoring='r2')
    rmse_scores = cross_val_score(model, X, y, cv=5,
scoring=rmse_scorer)

    print(f"{name} Cross-Validation:")
    print(f"  Mean R²: {np.mean(r2_scores):.5f} (+/-
{np.std(r2_scores):.5f})")
    print(f"  Mean RMSE: {np.mean(rmse_scores):.5f} (+/-
{np.std(rmse_scores):.5f})\n")
```

```
Linear Regression Model Evaluation:
  MAE: 0.00055
  MSE: 0.00000
  RMSE: 0.00062
  R²: 0.99757

Random Forest Model Evaluation:
  MAE: 0.00164
  MSE: 0.00000
  RMSE: 0.00201
  R²: 0.97417

Support Vector Regression (SVR) Model Evaluation:
  MAE: 0.00408
  MSE: 0.00002
  RMSE: 0.00481
  R²: 0.85260
```

```
XGBoost Model Evaluation:
  MAE: 0.00384
  MSE: 0.00002
  RMSE: 0.00426
  R²: 0.88432

Decision Tree Model Evaluation:
  MAE: 0.00493
  MSE: 0.00003
  RMSE: 0.00523
  R²: 0.82584

Bayesian Ridge Model Evaluation:
  MAE: 0.00043
  MSE: 0.00000
  RMSE: 0.00049
  R²: 0.99849

Neural Network (ANN) Model Evaluation:
  MAE: 0.55445
  MSE: 0.30743
  RMSE: 0.55447
  R²: -1955.22938


Cross-Validation Results:

Linear Regression Cross-Validation:
  Mean R²: 0.99048 (+/- 0.00860)
  Mean RMSE: 0.00066 (+/- 0.00011)

Random Forest Cross-Validation:
  Mean R²: 0.69799 (+/- 0.19836)
  Mean RMSE: 0.00522 (+/- 0.00254)

Support Vector Regression (SVR) Cross-Validation:
  Mean R²: -0.13739 (+/- 0.19364)
  Mean RMSE: 0.01059 (+/- 0.00572)

XGBoost Cross-Validation:
  Mean R²: 0.75054 (+/- 0.26708)
  Mean RMSE: 0.00422 (+/- 0.00208)

Decision Tree Cross-Validation:
  Mean R²: 0.50331 (+/- 0.35031)
  Mean RMSE: 0.00688 (+/- 0.00434)

Bayesian Ridge Cross-Validation:
  Mean R²: 0.99028 (+/- 0.00858)
  Mean RMSE: 0.00066 (+/- 0.00012)
```

```
Neural Network (ANN) Cross-Validation:
  Mean R²: -8747.55566 (+/- 10485.55622)
  Mean RMSE: 0.55510 (+/- 0.00400)
```

```python
import pandas as pd
import numpy as np
import seaborn as sns
import matplotlib.pyplot as plt
from sklearn.model_selection import train_test_split, cross_val_score
from sklearn.linear_model import LinearRegression, BayesianRidge
from sklearn.ensemble import RandomForestRegressor
from sklearn.svm import SVR
from xgboost import XGBRegressor
from sklearn.tree import DecisionTreeRegressor
from sklearn.metrics import mean_absolute_error, mean_squared_error,
r2_score

# Load the dataset
data = {
    "Initial Weight (g)": [77.700, 77.710, 77.705, 77.720, 77.690,
77.730, 77.698, 77.705, 77.708, 77.712,
                            80.640, 80.650, 80.645, 80.660, 80.635,
80.670, 80.638, 80.639, 80.642, 80.647],
    "Final Weight (g)": [77.388, 77.396, 77.396, 77.402, 77.379,
77.410, 77.391, 77.392, 77.393, 77.396,
                          80.323, 80.331, 80.329, 80.338, 80.318,
80.350, 80.319, 80.321, 80.322, 80.328],
    "Total Weight Loss (g)": [0.312, 0.314, 0.309, 0.318, 0.311,
0.320, 0.307, 0.313, 0.315, 0.316,
                               0.317, 0.319, 0.316, 0.322, 0.317,
0.320, 0.319, 0.318, 0.320, 0.319],
    "Corrosion Rate (mm/year)": [1.21451, 1.22193, 1.20377, 1.23803,
1.21092, 1.24663, 1.19436, 1.21822,
                                  1.22564, 1.22933, 1.23392, 1.24163,
1.22959, 1.25439, 1.23392, 1.24644,
                                  1.24163, 1.23730, 1.24644, 1.24163]
}

df = pd.DataFrame(data)

# Features and target
X = df[["Initial Weight (g)", "Final Weight (g)", "Total Weight Loss
(g)"]]
y = df["Corrosion Rate (mm/year)"]

# Train-test split
X_train, X_test, y_train, y_test = train_test_split(X, y,
test_size=0.2, random_state=42)
```

```python
# Define models
models = {
    "Linear Regression": LinearRegression(),
    "Random Forest": RandomForestRegressor(random_state=42,
n_estimators=100, max_depth=5),
    "Support Vector Regression (SVR)": SVR(kernel="rbf", C=10,
epsilon=0.01),
    "XGBoost": XGBRegressor(objective="reg:squarederror",
n_estimators=100, learning_rate=0.1, max_depth=3),
    "Decision Tree": DecisionTreeRegressor(random_state=42,
max_depth=5),
    "Bayesian Ridge": BayesianRidge()
}

# Store results
results = {}

# Function to evaluate models
def evaluate_model(name, model, X_train, X_test, y_train, y_test):
    model.fit(X_train, y_train)
    y_pred = model.predict(X_test)

    mae = mean_absolute_error(y_test, y_pred)
    mse = mean_squared_error(y_test, y_pred)
    rmse = np.sqrt(mse)
    r2 = r2_score(y_test, y_pred)

    # Store results
    results[name] = {"MAE": mae, "RMSE": rmse, "R²": r2, "y_pred":
y_pred}

    return model  # Return trained model for cross-validation

# Train and evaluate each model
trained_models = {}
for name, model in models.items():
    trained_models[name] = evaluate_model(name, model, X_train,
X_test, y_train, y_test)

# Perform Cross-validation
def rmse_scorer(estimator, X, y):
    y_pred = estimator.predict(X)
    return np.sqrt(mean_squared_error(y, y_pred))

cv_results = {}

for name, model in trained_models.items():
    r2_scores = cross_val_score(model, X, y, cv=5, scoring='r2')
    rmse_scores = cross_val_score(model, X, y, cv=5,
```

```
scoring=rmse_scorer)

    cv_results[name] = {
        "Mean R²": np.mean(r2_scores),
        "R² Std. Dev.": np.std(r2_scores),
        "Mean RMSE": np.mean(rmse_scores),
        "RMSE Std. Dev.": np.std(rmse_scores)
    }

# Convert results to DataFrame
df_results = pd.DataFrame(results).T
df_cv_results = pd.DataFrame(cv_results).T

# Combine results into final table
df_final = pd.concat([df_results, df_cv_results], axis=1)

# Display results
print("\nFinal Model Performance Comparison:")
print(df_final)

# Correlation Matrix Heatmap
plt.figure(figsize=(8,6))
sns.heatmap(df.corr(), annot=True, cmap="coolwarm", fmt=".2f")
plt.title("Feature Correlation Heatmap")
plt.show()

# Model Comparison - Bar Plot
df_final[["MAE", "RMSE", "R²"]].plot(kind="bar", figsize=(10,6))
plt.title("Model Performance Comparison")
plt.ylabel("Metric Value")
plt.xticks(rotation=45)
plt.legend(loc="best")
plt.show()

# Scatter Plot - Actual vs Predicted
plt.figure(figsize=(12, 6))
for name, model in trained_models.items():
    plt.scatter(y_test, results[name]["y_pred"], label=name,
alpha=0.6)
plt.plot([min(y_test), max(y_test)], [min(y_test), max(y_test)],
linestyle="--", color="black")
plt.xlabel("Actual Corrosion Rate")
plt.ylabel("Predicted Corrosion Rate")
plt.legend()
plt.title("Actual vs Predicted Corrosion Rates")
plt.show()

# Residual Plots
fig, ax = plt.subplots(3, 2, figsize=(12, 12))
ax = ax.flatten()
```

```python
for i, (name, model) in enumerate(trained_models.items()):
    residuals = y_test - results[name]["y_pred"]
    sns.histplot(residuals, kde=True, ax=ax[i])
    ax[i].set_title(f"Residual Plot: {name}")
plt.tight_layout()
plt.show()
```

```
Final Model Performance Comparison:
                                     MAE      RMSE        R²  \
Linear Regression               0.000552  0.000618  0.997573   
Random Forest                    0.00164  0.002015  0.974169   
Support Vector Regression (SVR)  0.004078  0.004813  0.852603   
XGBoost                         0.003838  0.004264   0.88432   
Decision Tree                   0.004925  0.005232  0.825842   
Bayesian Ridge                   0.00043  0.000487  0.998491   


y_pred  \
Linear Regression               [1.2143126647949218,
1.237799755859375, 1.2454...
Random Forest                   [1.2134271000000014,
1.2372193999999987, 1.249...
Support Vector Regression (SVR)  [1.2209806209191738,
1.2395460981821325, 1.239...
XGBoost                              [1.2110914, 1.2387586,
1.2530992, 1.2257457]
Decision Tree                                [1.21822, 1.24163,
1.25439, 1.22564]
Bayesian Ridge                  [1.2144454890184682,
1.2379264213517431, 1.245...

                                 Mean R²  R² Std. Dev.  Mean RMSE  \
Linear Regression               0.990478      0.008602   0.000656   
Random Forest                   0.697987      0.198364   0.005221   
Support Vector Regression (SVR) -0.137387      0.193636   0.010593   
XGBoost                         0.750537      0.267083   0.004222   
Decision Tree                   0.503312      0.350312   0.006884   
Bayesian Ridge                  0.990276      0.008582   0.000662   

                                RMSE Std. Dev.
Linear Regression                     0.000112
Random Forest                         0.002538
Support Vector Regression (SVR)       0.005723
XGBoost                               0.002080
Decision Tree                         0.004341
Bayesian Ridge                        0.000116
```

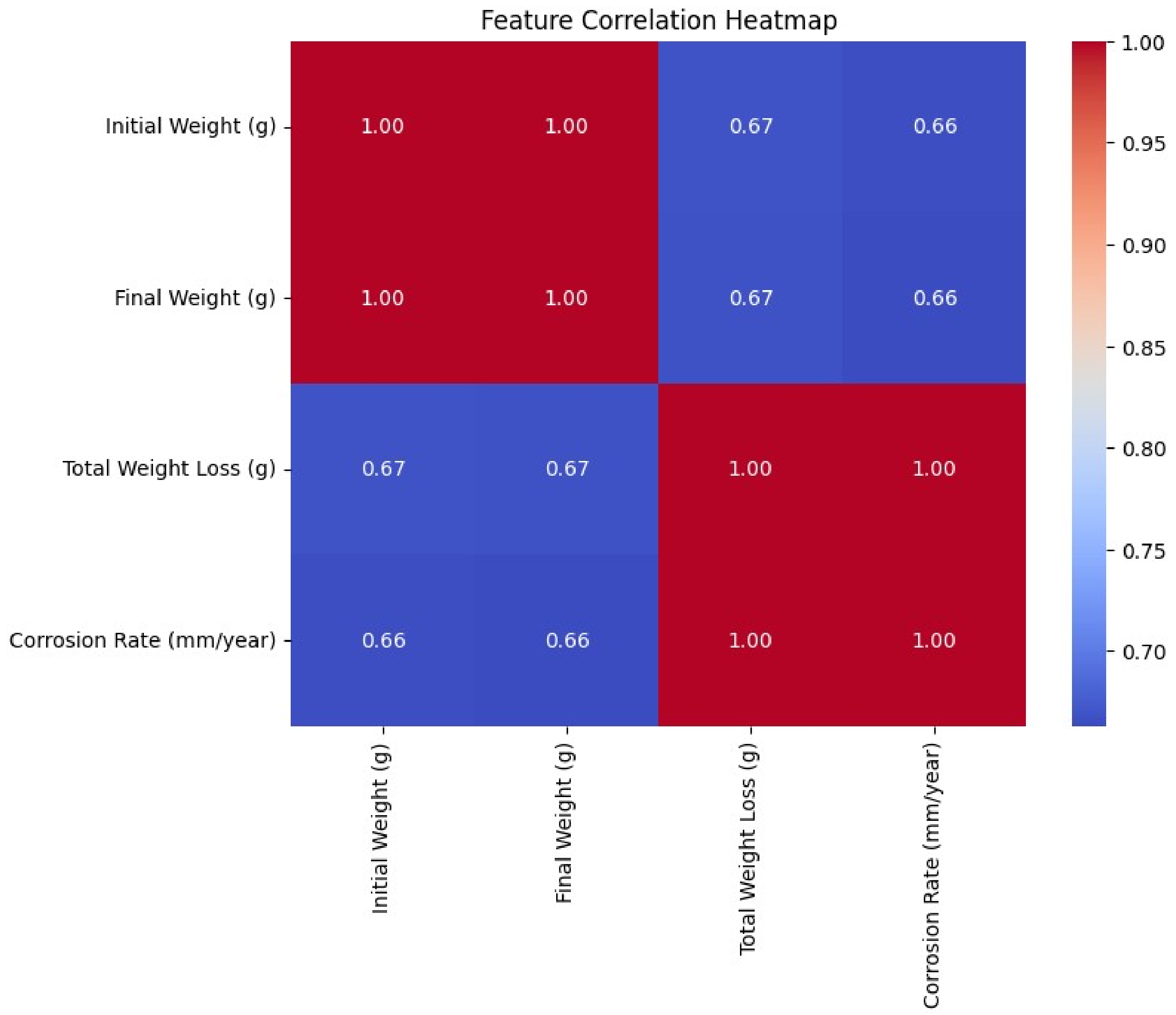
Feature Correlation Heatmap
Initial Weight (g)
Final Weight (g)
Total Weight Loss (g)
Corrosion Rate (mm/year)
1.00
1.00
0.67
0.66
1.00
1.00
0.67
0.66
0.67
0.67
1.00
1.00
0.66
0.66
1.00
1.00
Initial Weight (g)
Final Weight (g)
Total Weight Loss (g)
Corrosion Rate (mm/year)
1.00
0.95
0.90
0.85
0.80
0.75
0.70

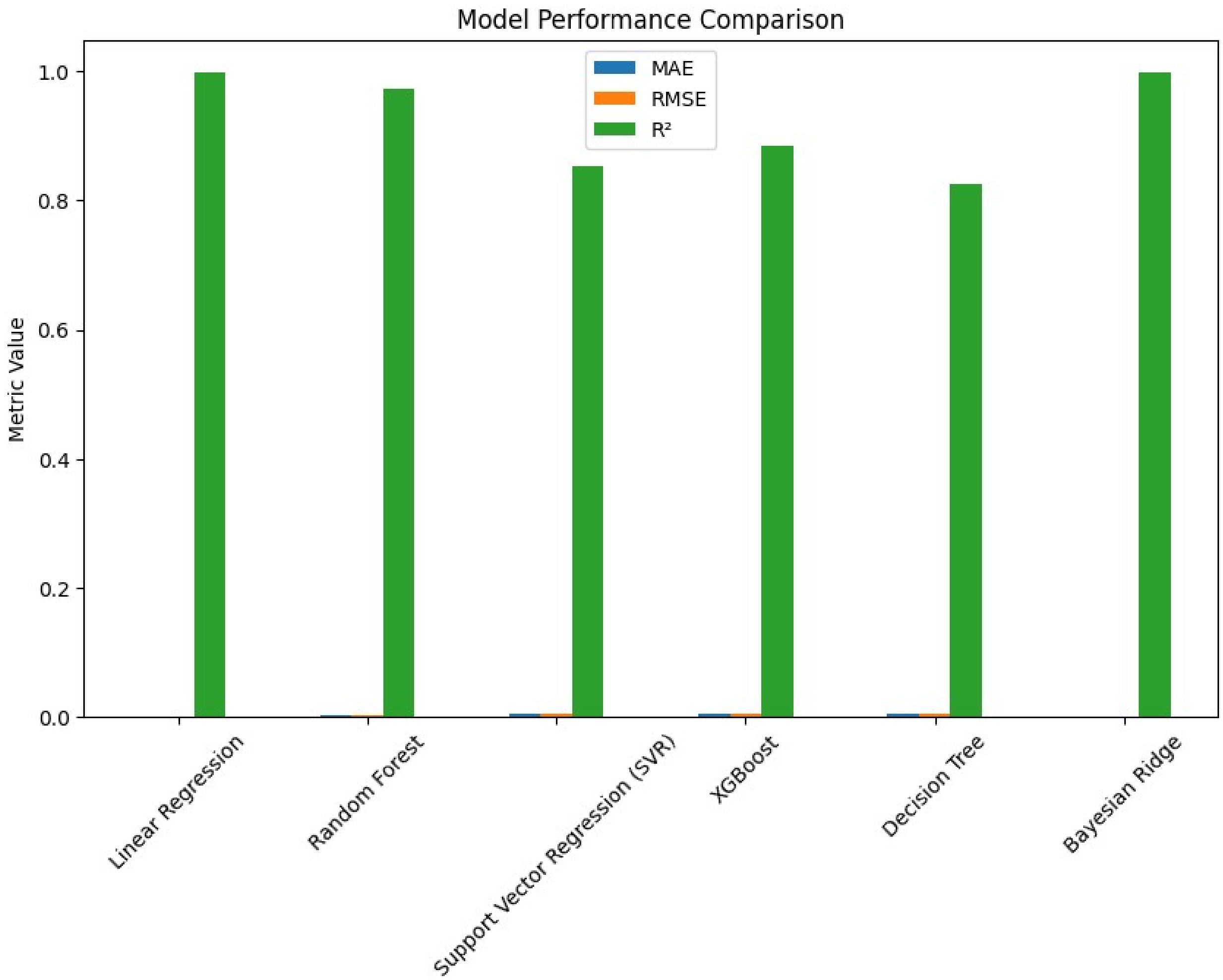
Model Performance Comparison
MAE
RMSE
R²
Metric Value
1.0
0.8
0.6
0.4
0.2
0.0
Linear Regression
Random Forest
Support Vector Regression (SVR)
XGBoost
Decision Tree
Bayesian Ridge

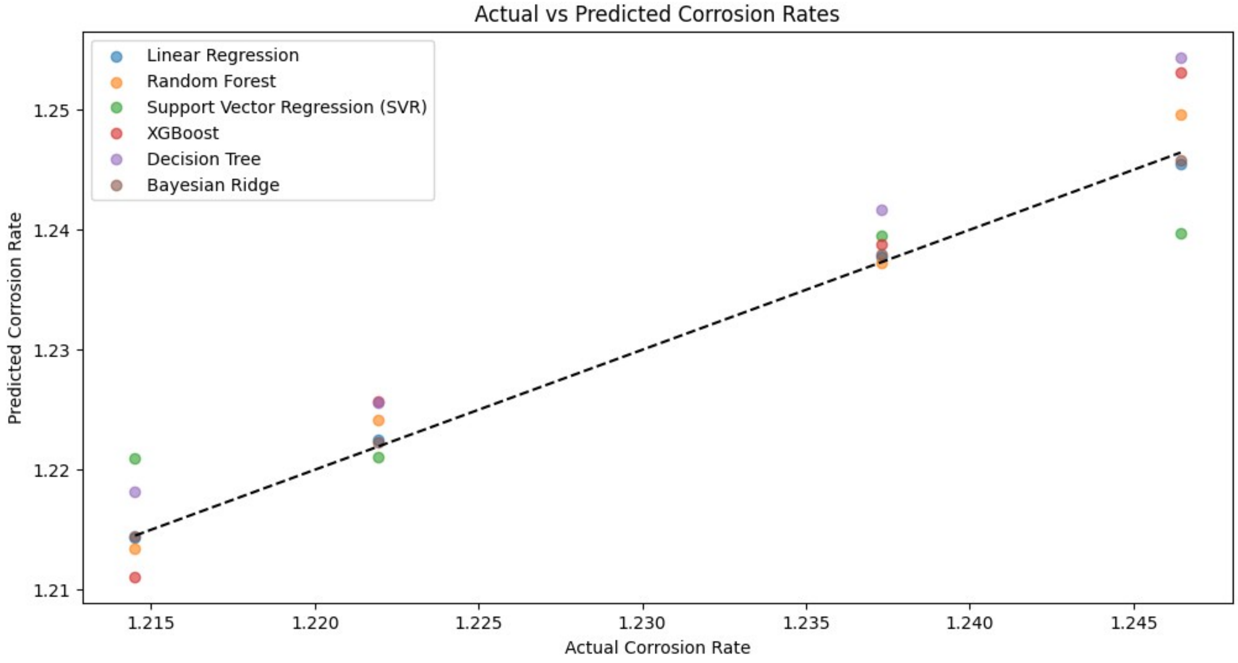
Actual vs Predicted Corrosion Rates
Linear Regression
Random Forest
Support Vector Regression (SVR)
XGBoost
Decision Tree
Bayesian Ridge
Predicted Corrosion Rate
1.25
1.24
1.23
1.22
1.21
1.215
1.220
1.225
1.230
1.235
1.240
1.245
Actual Corrosion Rate

```
/usr/local/lib/python3.10/dist-packages/seaborn/_oldcore.py:1119:
FutureWarning: use_inf_as_na option is deprecated and will be removed
in a future version. Convert inf values to NaN before operating
instead.
  with pd.option_context('mode.use_inf_as_na', True):
/usr/local/lib/python3.10/dist-packages/seaborn/_oldcore.py:1119:
FutureWarning: use_inf_as_na option is deprecated and will be removed
in a future version. Convert inf values to NaN before operating
instead.
  with pd.option_context('mode.use_inf_as_na', True):
/usr/local/lib/python3.10/dist-packages/seaborn/_oldcore.py:1119:
FutureWarning: use_inf_as_na option is deprecated and will be removed
in a future version. Convert inf values to NaN before operating
instead.
  with pd.option_context('mode.use_inf_as_na', True):
/usr/local/lib/python3.10/dist-packages/seaborn/_oldcore.py:1119:
FutureWarning: use_inf_as_na option is deprecated and will be removed
in a future version. Convert inf values to NaN before operating
instead.
  with pd.option_context('mode.use_inf_as_na', True):
/usr/local/lib/python3.10/dist-packages/seaborn/_oldcore.py:1119:
FutureWarning: use_inf_as_na option is deprecated and will be removed
in a future version. Convert inf values to NaN before operating
instead.
  with pd.option_context('mode.use_inf_as_na', True):
/usr/local/lib/python3.10/dist-packages/seaborn/_oldcore.py:1119:
FutureWarning: use_inf_as_na option is deprecated and will be removed
in a future version. Convert inf values to NaN before operating
instead.
  with pd.option_context('mode.use_inf_as_na', True):
```

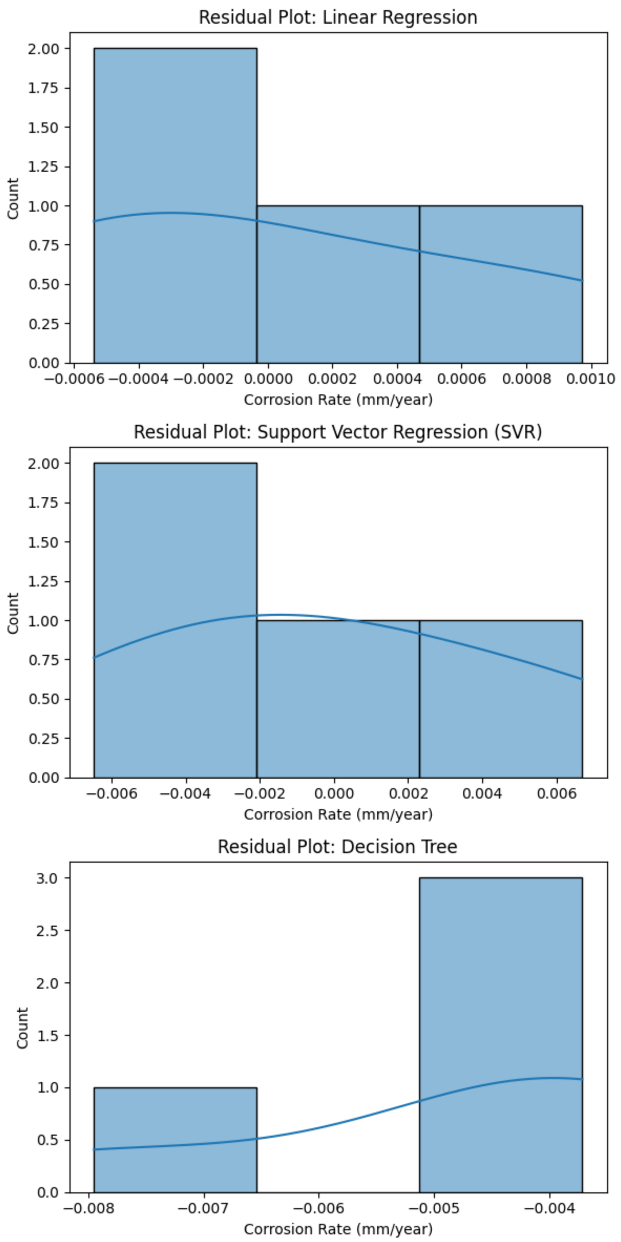


```
import matplotlib.pyplot as plt

# Extract necessary metrics
mae_values = df_final["MAE"]
rmse_values = df_final["RMSE"]
r2_values = df_final["R²"]

# Plot with dual axes
fig, ax1 = plt.subplots(figsize=(10,6))

# Primary y-axis (left) for MAE & RMSE
ax1.set_xlabel("Models")
```

```
ax1.set_ylabel("MAE / RMSE", color="tab:blue")
ax1.bar(df_final.index, mae_values, color="blue", label="MAE",
alpha=0.6)
ax1.bar(df_final.index, rmse_values, color="orange", label="RMSE",
alpha=0.6, bottom=mae_values)
ax1.tick_params(axis='y', labelcolor="tab:blue")

# Secondary y-axis (right) for R²
ax2 = ax1.twinx()
ax2.set_ylabel("R² Score", color="tab:green")
ax2.plot(df_final.index, r2_values, color="green", marker="o",
linestyle="dashed", linewidth=2, label="R²")
ax2.tick_params(axis='y', labelcolor="tab:green")

# Labels & Legends
fig.tight_layout()
ax1.set_xticklabels(df_final.index, rotation=45)
ax1.legend(loc="upper left")
ax2.legend(loc="upper right")
plt.title("Model Performance Comparison (Dual Y-Axis)")

plt.show()
```

```
<ipython-input-8-e110880dba54>:26: UserWarning: FixedFormatter should
only be used together with FixedLocator
  ax1.set_xticklabels(df_final.index, rotation=45)
```

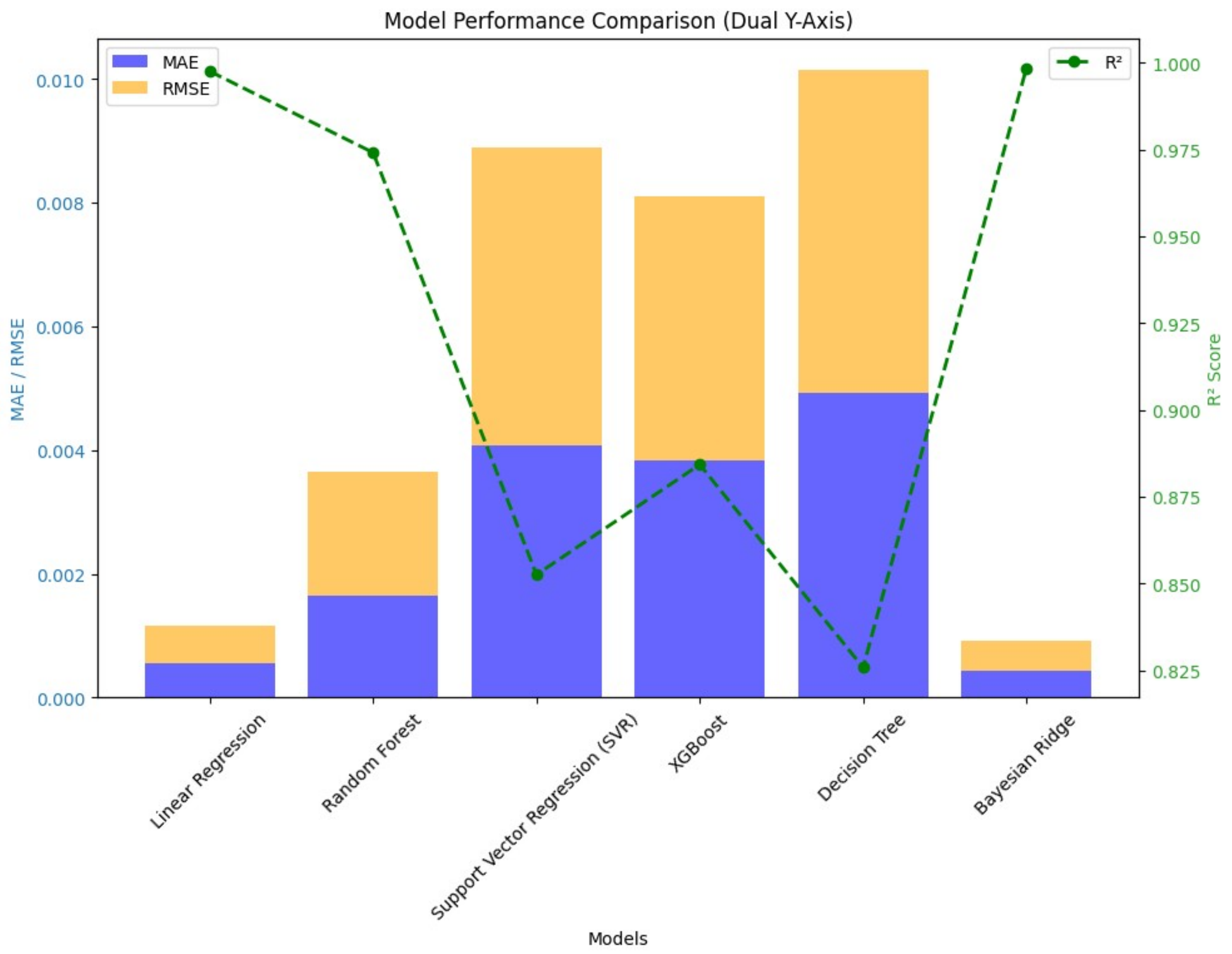
Model Performance Comparison (Dual Y-Axis)
MAE
RMSE
R²
MAE / RMSE
R² Score
0.000
0.002
0.004
0.006
0.008
0.010
0.825
0.850
0.875
0.900
0.925
0.950
0.975
1.000
Linear Regression
Random Forest
Support Vector Regression (SVR)
XGBoost
Decision Tree
Bayesian Ridge
Models
